# Supplementary material for: De Novo Hybrid Assembly Unveils Multi-Chromosomal Mitochondrial Genomes in Ludwigia Species, Highlighting Genomic Recombination, Gene Transfer, and RNA Editing Events
Source: Int J Mol Sci. 2024 Jul 2;25(13):7283. doi: 10.3390/ijms25137283 (PMC11242644; doi:10.3390/ijms25137283)
Supplement: Supplementary file 1 [file ijms-25-07283-s001.zip › Supplementary_figures.pdf]

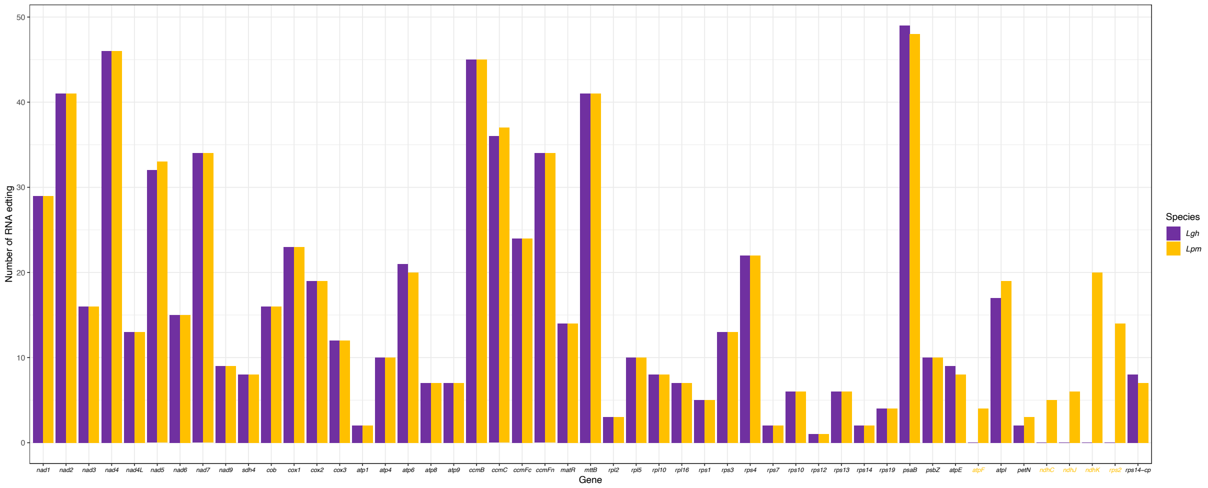

Figure S1. Number of RNA editing per gene, identified by Deepred-Mt, in *Ludwigia grandiflora* subsp. *hexapetala* (*Lgh* in purple) and *Ludwigia peploides* subsp. *montevidensis* (*Lpm* in yellow) mitogenomes.

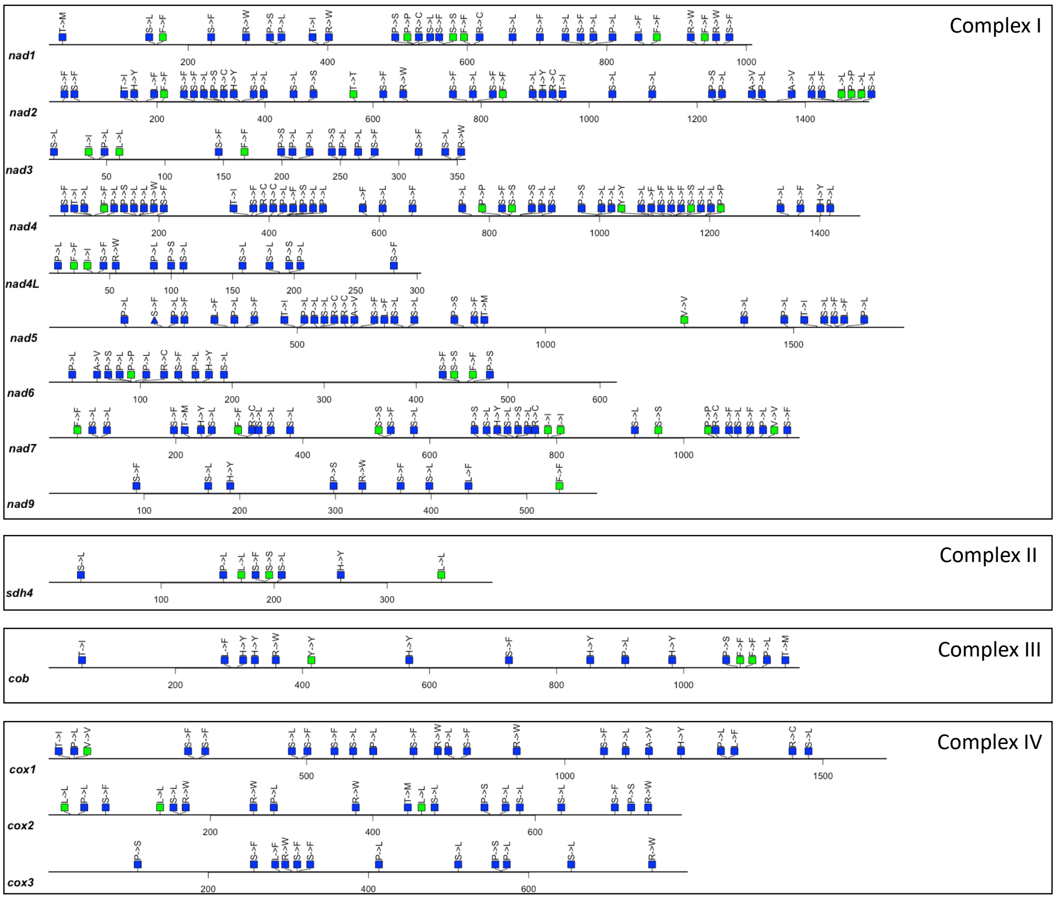

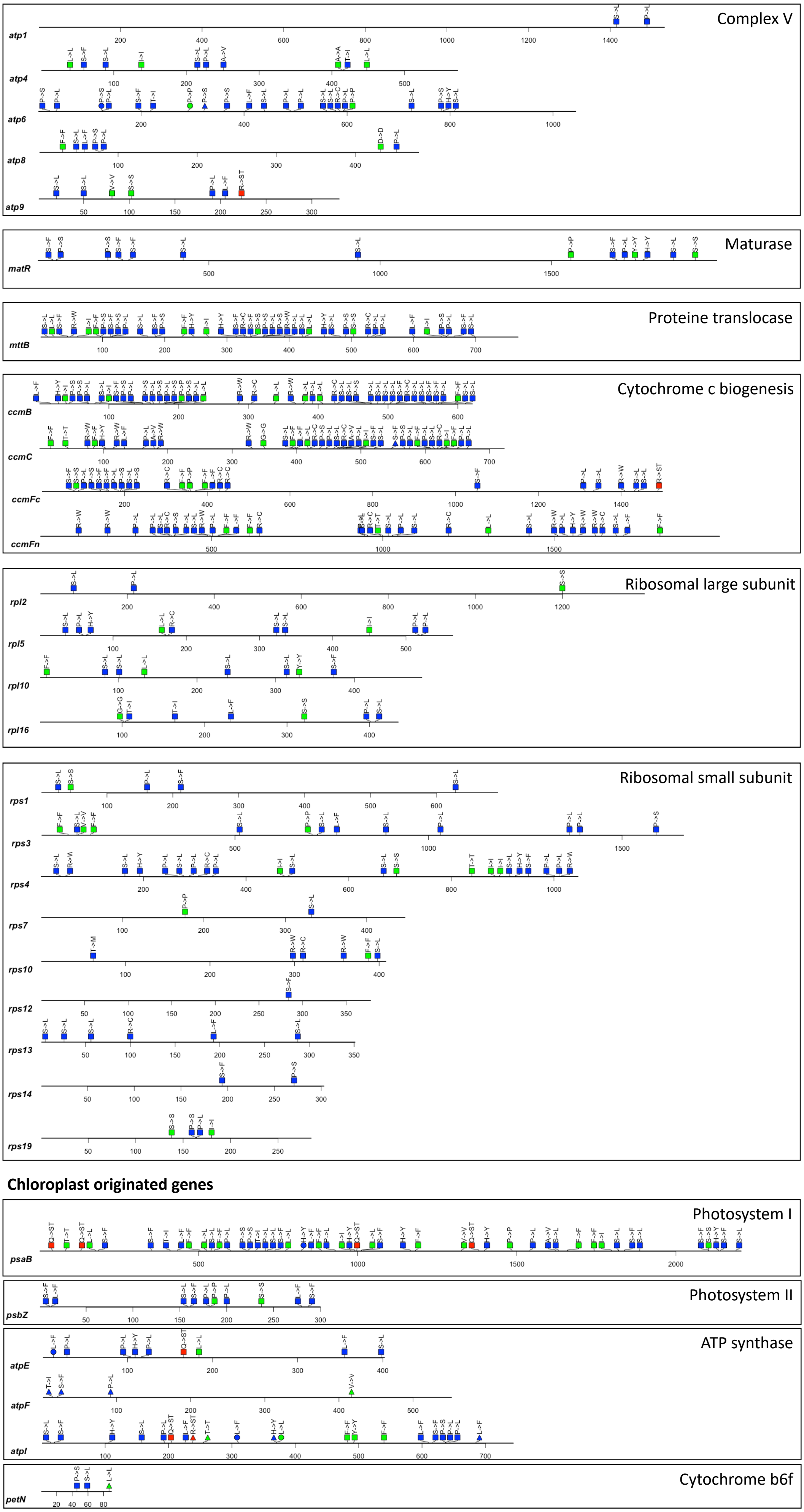

Complex V
atp1
atp4
atp6
atp8
atp9
Maturase
matR
Proteine translocase
mttB
Cytochrome c biogenesis
ccmB
ccmC
ccmFc
ccmFn
Ribosomal large subunit
rpl2
rpl5
rpl10
rpl16
Ribosomal small subunit
rps1
rps3
rps4
rps7
rps10
rps12
rps13
rps14
rps19
Chloroplast originated genes
Photosystem I
psaB
Photosystem II
psbZ
ATP synthase
atpE
atpF
atpI
Cytochrome b6f
petN

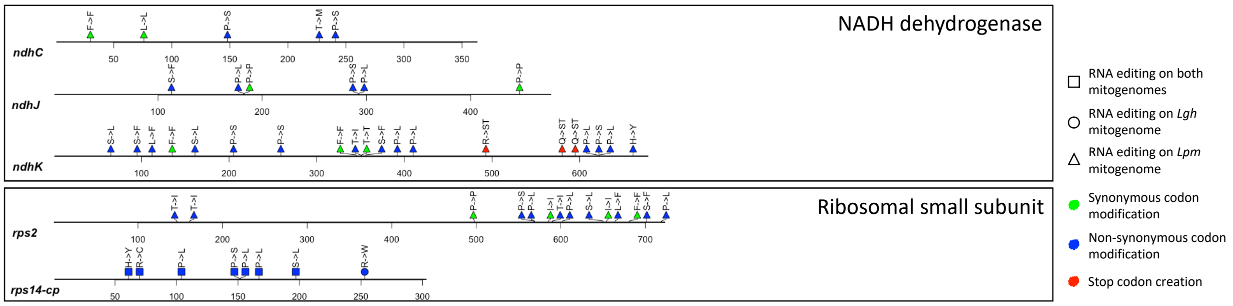

Figure S2. Representation of RNA editing sites in *Ludwigia grandiflora* subsp. *hexapetala* (*Lgh*) and *Ludwigia peploides* subsp. *montevidensis* (*Lpm*) mitogenomes protein-coding genes.

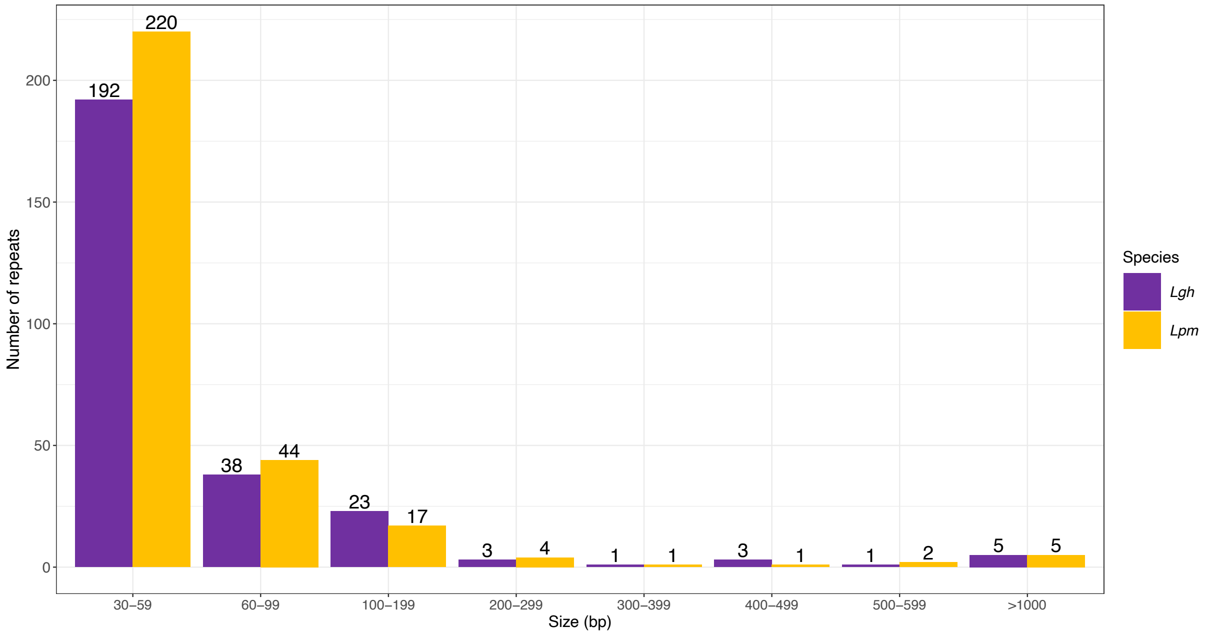

Figure S3. Number of non-tandem repeats based on their size in *Ludwigia grandiflora* subsp. *hexapetala* (*Lgh*) and *Ludwigia peploides* subsp. *montevidensis* (*Lpm*) mitogenomes.

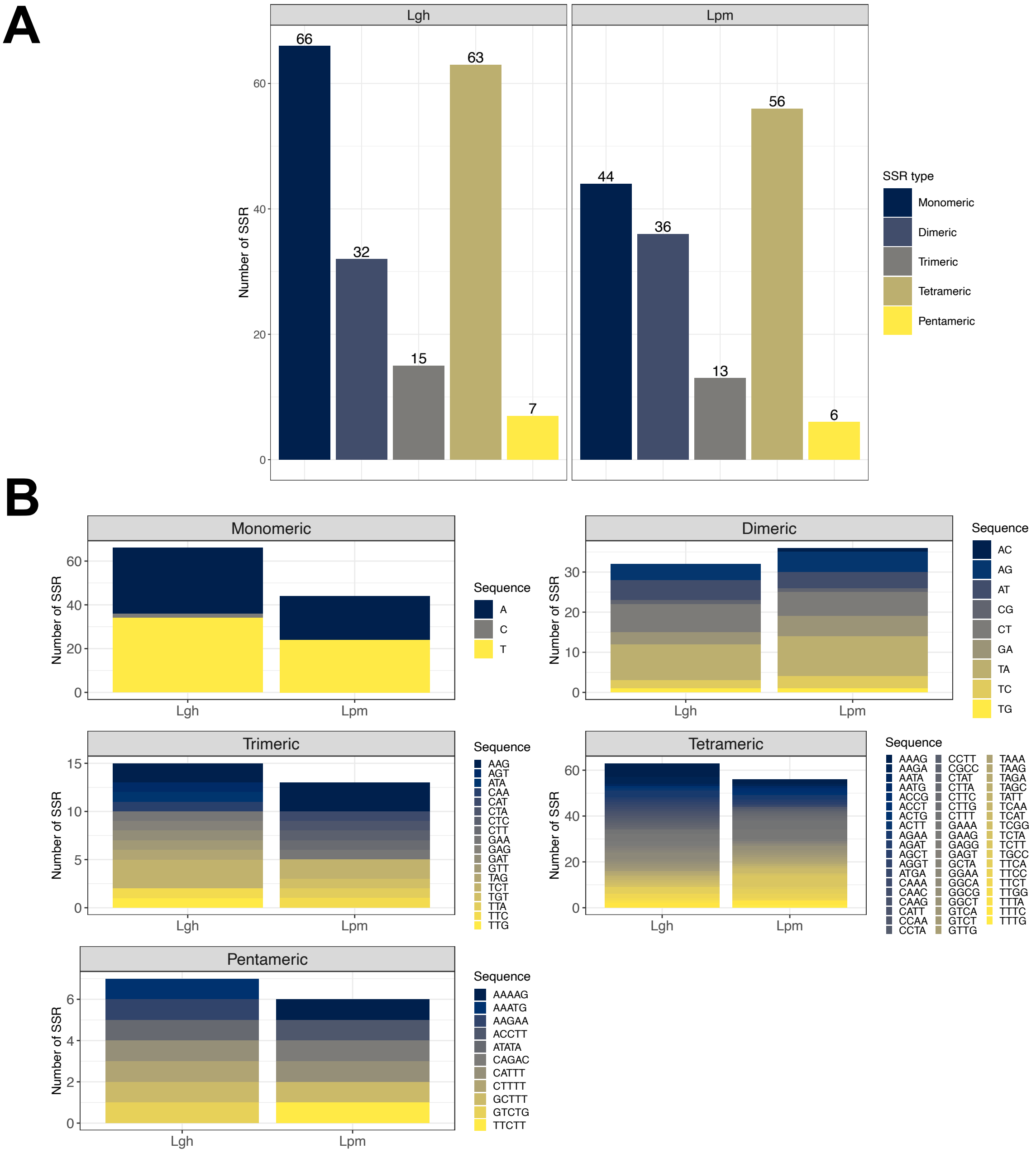

Figure S4. Simple Sequence Repeats (SSR) in *Ludwigia grandiflora* subsp. *hexapetala* (*Lgh*) and *Ludwigia peploides* subsp. *montevidensis* (*Lpm*) mitogenomes. A: Number of each SSR type. B: Detailed compositions of each SSR type.
