# Supplementary material for: De Novo Hybrid Assembly Unveils Multi-Chromosomal Mitochondrial Genomes in Ludwigia Species, Highlighting Genomic Recombination, Gene Transfer, and RNA Editing Events
Source: Int J Mol Sci. 2024 Jul 2;25(13):7283. doi: 10.3390/ijms25137283 (PMC11242644; doi:10.3390/ijms25137283)
Supplement: Supplementary file 1 [file ijms-25-07283-s001.zip › Supplementary_tables.pdf]

Table S1: Comparison of Flye, Megahit and SPAdes assembly performances using *Ludwigia grandiflora* subsp. *hexapetala* mitogenomes M1 and M2 as the reference, evaluated with QUAST. All statistics are derived from contigs with a size ≥ 500 bp, unless otherwise specified (e.g., "# contigs (≥ 0 bp)" and "Total length (≥ 0 bp)" encompass all contigs).

**Alignment-based statistics**	**Flye**	**Megahit**	**SPAdes**
Genome fraction (%)	92.42	89.066	94.922
Duplication ratio	1.003	1.007	1.038
Largest alignment	105 008	38 614	191 601
Total aligned length	663 802	642 161	704 311
NGA50	53 304	15 088	80 045
LGA50	5	15	3
**Misassemblies**			
# misassemblies	0	0	3
Misassembled contigs length	0	0	439 030
**Per base quality**			
# mismatches per 100 kbp	70.76	46.22	146.93
# mismatches	473	296	1041
# indels per 100 kbp	12.91	5.44	34.29
# indels	87	35	244
# indels (≤ 5 bp)	78	32	208
# indels (> 5 bp)	9	3	36
Indels length	226	91	1012
**Statistics without reference**			
# contigs (≥ 0 bp)	39	179	35
# contigs (≥ 500 bp)	37	70	20
# contigs (≥ 1000 bp)	22	67	13
# contigs (≥ 5000 bp)	16	35	8
# contigs (≥ 10000 bp)	13	18	7
# contigs (≥ 25000 bp)	11	7	5
# contigs (≥ 50000 bp)	6	0	5
Largest contig	105 008	43 938	191 601
Total length (≥ 0 bp)	661 029	681 941	812 440
Total length (≥ 500 bp)	660 613	648 165	806 438
Total length (≥ 1000 bp)	650 062	646 177	801 733
Total length (≥ 5000 bp)	641 311	550 392	789 051
Total length (≥ 10000 bp)	619 455	414 136	780 610
Total length (≥ 25000 bp)	596 263	214 902	752 738
Total length (≥ 50000 bp)	420 228	0	752 738
GC (%)	45.1	45.24	43.79

Table S2: Comparisons of locally collinear blocks (LCBs) identified by Progressive-Mauve between *Ludwigia grandiflora* subsp. *hexapetala* (*Lgh*) and *Ludwigia peploides* subsp. *montevidensis* (*Lpm*) mitogenomes.

LCB name	Length in *Lpm* (bp)	Length in *Lgh* (bp)	Identity %
	**Molecule M2**		
LCB0	167,000	166,796	99.5
	**Molecule M1**		
LCB1	97,795	97,852	99.7
LCB2	1,046	1,046	99.1
LCB3	48,239	48,292	99
LCB4	50,794	50,805	99.6
LCB5	152,180	152,309	99.7
LCB6	12,325	12,514	86.6
LCB7	83,140	83,214	99.8
LCB8	45,749	45,778	99.9

Table S3: Chloroplast insertions in *Ludwigia grandiflora* subsp. *hexapetala* (*Lgh*) and *Ludwigia peploides* subsp. *montevidensis* (*Lpm*) mitogenomes. Genes are color-coded as follows: red for pseudogenes, cyan for pseudogenes due to RNA editing, orange for fragment genes, black for complete protein coding genes, purple for tRNA genes, and magenta for rRNA genes. Locally Collinear Blocks (LCBs) correspond to conserved regions between *Lgh* and *Lpm* mitogenomes, identified by Progressive-Mauve. The term "interLCB" indicates that the sequence is situated between two LCBs.

LCB	Coordinates	Length (bp)	Identity %	Genes
Ludwigia grandiflora* subsp. *hexapetala				
**Molecule M2**				
LCB0	17,289-17,785	497	100	*rps7**
LCB0	17,776-18,317	542	95.8	Δ*rps12*
LCB0	26,101-26,343	243	88.5	Δ*rps12*
LCB0	45,455-45,982	528	92.1	Δ*ndhB*
LCB0	158,507-166,557	8051	98.4	*atpA**, *atpF**, *atpH**, *atpI**, *rps2**, Δ*rpoC2*
**Molecule M1**				
LCB1	15-379	365	97.1	Δ*paf1*
LCB3	37,376-37,737	362	67.8	Δ*petB*
LCB3	57,951-62,745	4795	99.9	Δ*psaA*, *psaB**, ***rps14***, ***trnM-CAU***, ***trnG-GCC***, ***psbZ***, ***trnS-UGA***
LCB4	103,876-110,040	6165	99.6	***trnV-GAC***, ***rrn16***, ***trnI-GAU***, ***trnA-UGC***, Δ*rrn23*
LCB4	112,517-113,116	600	100	Δ*ndhA*
LCB4	115,795-115,940	146	94.6	Δ*psaA*
LCB4	116,140-116,292	153	81	Δ*accD*
LCB5	185,591-191,050	5460	89.3	*ndhJ**, *ndhK**, *ndhC**, ***trnV-UAC***, ***trnM-CAU***, *atpE**, Δ*atpB*
LCB5	206,734-206,941	208	86.5	Δ*atpB*
LCB5	215,323-215,452	130	77.9	Δ*ycf2*
LCB5	215,984-216,324	341	83.8	***trnP-UGG***, ***trnW-CCA***
LCB5	245,287-245,855	569	74.7	***trnD-GUC***
LCB5	246,325-246,633	309	93.5	***petN***
LCB5	246,921-248,135	1215	89.7	Δ*psbD*, Δ*psbC*
LCB7	297,606-301,261	3656	100	Δ*rrn23*, ***rrn4.5***, ***rrn5***, ***trnR-ACG***, ***trnN-GUU***, Δ*ycf1*
LCB7	353,346-353,464	119	84.9	
LCB7	377,423-378,440	1018	73.7	***trnS-GGA***
LCB1	491,174-491,425	252	100	Δ*rpl2*
LCB1	534,557-542,192	7636	92.8	Δ*ycf15*, *ycf2**, ***trnM-CAU***
Ludwigia peploides* subsp. *montevidensis				
**Molecule M2**				
LCB0	17,286-17,783	498	97.4	*rps7**
LCB0	17,774-18,315	542	93.9	Δ*rps12*
LCB0	26,097-26,339	243	88.9	Δ*rps12*
LCB0	45,440-45,967	528	100	Δ*ndhB*
LCB0	158,507-166,557	8051	98.4	*atpA**, ***atpF***, *atpH**, *atpI**, ***rps2***, Δ*rpoC2*
**Molecule M1**				
LCB1	1-382	382	88.7	Δ*paf1*
LCB3	379,206-379,537	332	68	Δ*petB*
LCB3	354,205-358,879	4675	99.9	Δ*psaA*, *psaB**, ***rps14***, ***trnM-CAU***, ***trnG-GCC***, ***psbZ***, ***trnS-UGA***
LCB4	415,927-422,111	6185	99.6	***trnV-GAC***, ***rrn16***, ***trnI-GAU***, ***trnA-UGC***, Δ*rrn23*
LCB4	412,854-413,449	596	96	Δ*ndhA*
LCB4	410,033-410,170	138	95.5	Δ*psaA*
LCB4	409,710-409,854	145	79.2	Δ*accD*
LCB5	204,896-210,306	5411	91.9	***ndhJ***, *ndhK**, ***ndhC***, ***trnV-UAC***, ***trnM-CAU***, *atpE**, Δ*atpB*
LCB5	189,010-189,217	208	86.1	Δ*atpB*
LCB5	180,503-180,632	130	78.7	Δ*ycf2*
LCB5	179,632-179,994	363	82.8	***trnP-UGG***, ***trnW-CCA***
LCB5	150,130-150,687	558	79.4	***trnD-GUC***
LCB5	149,349-149,657	309	94.8	***petN***
LCB5	147,831-149,052	1222	93.5	Δ*psbD*, Δ*psbC*
LCB7	95,009-98,538	3530	99.8	Δ*rrn23*, ***rrn4.5***, ***rrn5***, ***trnR-ACG***, ***trnN-GUU***, Δ*ycf1*
LCB7	42,831-42,965	135	86.7	
LCB7	17,297-18,884	1588	65.1	***trnS-GGA***
LCB1	501,949-502,200	252	96	Δ*rpl2*
LCB1	545,340-553,004	7665	93	Δ*ycf15*, *ycf2**, ***trnM-CAU***
interLCB	335,484-335,571	88	85.2	Δ*accD*

Table S4: RNA editing sites in *Ludwigia grandiflora* subsp. *hexapetala* (*Lgh*) and *Ludwigia peploides* subsp. *montevidensis* (*Lpm*) mitogenomes protein-coding genes identified by Deepred-Mt. Yellow and purple lines represent RNA editing unique to *Lpm* and *Lgh*, respectively. Amino acid changes in blue and green indicate non-synonymous and synonymous modifications, respectively. Changes in red signify the appearance of a stop codon.

Gene	Position	Base codon	New codon	Aminoacid change	Probability
nad1	20	ACG	ATG	T->M	0.963
nad1	152	TCG	TTG	S->L	0.76
nad1	156	TTC	TTT	F->F	0.685
nad1	233	TCC	TTC	S->F	0.999
nad1	283	CGG	TGG	R->W	0.999
nad1	325	CCG	TCG	P->S	0.995
nad1	326	CCG	CTG	P->L	1
nad1	386	ACA	ATA	T->I	0.998
nad1	394	CGG	TGG	R->W	0.994
nad1	520	CCC	TCC	P->S	0.997
nad1	522	CCC	CCT	P->P	0.989
nad1	523	CGT	TGT	R->C	0.995
nad1	530	TCG	TTG	S->L	1
nad1	566	TCC	TTC	S->F	0.997
nad1	567	TCC	TCT	S->S	0.996
nad1	603	TTC	TTT	F->F	1
nad1	610	CGT	TGT	R->C	1
nad1	665	TCA	TTA	S->L	0.999
nad1	704	TCT	TTT	S->F	1
nad1	764	TCG	TTG	S->L	0.826
nad1	770	TCT	TTT	S->F	0.937
nad1	773	CCA	CTA	P->L	0.993
nad1	785	CCG	CTG	P->L	0.995
nad1	853	CTC	TTC	L->F	0.99
nad1	864	TTC	TTT	F->F	0.671
nad1	928	CGG	TGG	R->W	0.999
nad1	939	TTC	TTT	F->F	0.558
nad1	958	CGG	TGG	R->W	0.999
nad1	968	TCT	TTT	S->F	0.871
nad2	26	TCC	TTC	S->F	0.996
nad2	56	TCC	TTC	S->F	0.999
nad2	155	ACT	ATT	T->I	0.687
nad2	163	CAT	TAT	H->Y	0.532
nad2	181	CTT	TTT	L->F	0.997
nad2	210	TTC	TTT	F->F	0.653
nad2	266	TCT	TTT	S->F	0.997
nad2	269	TCC	TTC	S->F	0.999
nad2	314	CCA	CTA	P->L	0.995
nad2	319	CCT	TCT	P->S	0.999
nad2	325	CGC	TGC	R->C	0.989
nad2	352	CAT	TAT	H->Y	0.997
nad2	359	TCA	TTA	S->L	0.995
nad2	386	CCT	CTT	P->L	0.999
nad2	455	TCG	TTG	S->L	0.999
nad2	481	CCC	TCC	P->S	0.997
nad2	567	ACC	ACT	T->T	0.697
nad2	620	TCT	TTT	S->F	0.996
nad2	664	CGG	TGG	R->W	0.656
nad2	688	CTA	TTA	L->L	0.511
nad2	773	TCT	TTT	S->F	0.999
nad2	785	TCA	TTA	S->L	0.966
nad2	794	TCT	TTT	S->F	0.998
nad2	840	TTC	TTT	F->F	0.538
nad2	905	CCT	CTT	P->L	0.999
nad2	905	CCT	CTT	P->L	0.997
nad2	913	CAT	TAT	H->Y	0.995
nad2	913	CAT	TAT	H->Y	0.993
nad2	943	CGT	TGT	R->C	1
nad2	947	ACT	ATT	T->I	0.997
nad2	1043	TCA	TTA	S->L	0.996
nad2	1112	TCG	TTG	S->L	0.999

nad2	1231	CCA	TCA	P->S	0.995
nad2	1232	CCA	CTA	P->L	0.683
nad2	1325	GCA	GTA	A->V	0.757
nad2	1328	CCG	CTG	P->L	0.846
nad2	1349	GCG	GTG	A->V	0.999
nad2	1451	TCA	TTA	S->L	0.986
nad2	1454	TCC	TTC	S->F	0.97
nad2	1459	CTA	TTA	L->L	0.962
nad2	1467	CCC	CCT	P->P	0.966
nad2	1474	CTG	TTG	L->L	0.76
nad2	1508	TCA	TTA	S->L	0.996
nad3	5	TCA	TTA	S->L	0.966
nad3	39	ATC	ATT	I->I	0.855
nad3	44	CCG	CTG	P->L	1
nad3	61	CTA	TTA	L->L	0.514
nad3	146	TCC	TTC	S->F	0.999
nad3	168	TTC	TTT	F->F	0.692
nad3	208	CCT	TCT	P->S	0.998
nad3	209	CCT	CTT	P->L	0.996
nad3	215	CCG	CTG	P->L	0.997
nad3	247	CCT	TCT	P->S	0.996
nad3	251	CCC	CTC	P->L	0.981
nad3	266	CCC	CTC	P->L	0.999
nad3	275	TCT	TTT	S->F	0.992
nad3	317	TCT	TTT	S->F	1
nad3	344	TCG	TTG	S->L	0.999
nad3	349	CGG	TGG	R->W	0.999
nad4	29	TCT	TTT	S->F	1
nad4	74	ACT	ATT	T->I	0.997
nad4	77	CCT	CTT	P->L	0.999
nad4	84	TTC	TTT	F->F	0.678
nad4	107	CCG	CTG	P->L	0.999
nad4	154	CCC	TCC	P->S	0.999
nad4	158	CCT	CTT	P->L	0.997
nad4	164	CCT	CTT	P->L	0.994
nad4	166	CGG	TGG	R->W	1
nad4	197	TCT	TTT	S->F	0.998
nad4	362	ACA	ATA	T->I	0.999
nad4	368	TCT	TTT	S->F	0.996
nad4	376	CGT	TGT	R->C	0.995
nad4	403	CGC	TGC	R->C	0.999
nad4	416	CCT	CTT	P->L	0.992
nad4	433	CTT	TTT	L->F	0.999
nad4	436	CCC	TCC	P->S	0.999
nad4	437	CCC	CTC	P->L	0.992
nad4	449	CCA	CTA	P->L	0.993
nad4	577	CTC	TTC	L->F	0.55
nad4	608	TCA	TTA	S->L	0.999
nad4	659	TCT	TTT	S->F	1
nad4	767	CCT	CTT	P->L	0.999
nad4	819	CCC	CCT	P->P	0.601
nad4	836	TCC	TTC	S->F	0.995
nad4	837	TCC	TCT	S->S	0.613
nad4	856	CCA	TCA	P->S	0.979
nad4	857	CCA	CTA	P->L	1
nad4	896	TCA	TTA	S->L	1
nad4	994	CCA	TCA	P->S	0.606
nad4	995	CCA	CTA	P->L	0.999
nad4	998	CCG	CTG	P->L	0.999
nad4	1089	TAC	TAT	Y->Y	0.906
nad4	1097	TCA	TTA	S->L	0.999
nad4	1117	CTC	TTC	L->F	0.997
nad4	1130	TCC	TTC	S->F	0.989
nad4	1136	TCT	TTT	S->F	0.998
nad4	1139	TCC	TTC	S->F	0.995
nad4	1140	TCC	TCT	S->S	0.923
nad4	1160	TCA	TTA	S->L	1
nad4	1193	CCC	CTC	P->L	0.999
nad4	1194	CCC	CCT	P->P	0.634
nad4	1343	CCA	CTA	P->L	0.995
nad4	1361	TCC	TTC	S->F	0.999
nad4	1402	CAC	TAC	H->Y	0.997
nad4	1418	CCG	CTG	P->L	0.998
nad4L	8	CCT	CTT	P->L	0.847
nad4L	21	TTC	TTT	F->F	0.573
nad4L	36	ATC	ATT	I->I	0.557
nad4L	41	TCT	TTT	S->F	0.994
nad4L	55	CGG	TGG	R->W	0.996

nad4L	86	CCT	CTT	P->L	0.996
nad4L	100	CCA	TCA	P->S	0.999
nad4L	110	TCA	TTA	S->L	0.998
nad4L	158	TCG	TTG	S->L	0.996
nad4L	188	TCA	TTA	S->L	1
nad4L	196	CCA	TCA	P->S	0.734
nad4L	197	CCA	CTA	P->L	1
nad4L	281	TCT	TTT	S->F	0.999
nad5	155	CCG	CTG	P->L	1
nad5	227	TCC	TTC	S->F	1
nad5	242	CCG	CTG	P->L	0.999
nad5	242	CCG	CTG	P->L	1
nad5	272	TCC	TTC	S->F	0.999
nad5	358	CTT	TTT	L->F	0.994
nad5	374	CCA	CTA	P->L	0.997
nad5	398	TCT	TTT	S->F	1
nad5	494	ACA	ATA	T->I	1
nad5	506	CCT	CTT	P->L	0.995
nad5	539	CCT	CTT	P->L	1
nad5	548	TCG	TTG	S->L	0.998
nad5	553	CGT	TGT	R->C	0.999
nad5	598	CGT	TGT	R->C	0.999
nad5	608	GCC	GTC	A->V	0.993
nad5	629	TCT	TTT	S->F	0.999
nad5	676	CTT	TTT	L->F	1
nad5	713	TCG	TTG	S->L	0.999
nad5	725	TCA	TTA	S->L	1
nad5	835	CCA	TCA	P->S	1
nad5	863	TCT	TTT	S->F	0.999
nad5	875	ACG	ATG	T->M	1
nad5	1281	GTC	GTT	V->V	0.577
nad5	1400	TCA	TTA	S->L	1
nad5	1487	CCC	CTC	P->L	0.999
nad5	1547	ACC	ATC	T->I	0.998
nad5	1577	TCA	TTA	S->L	0.996
nad5	1586	TCT	TTT	S->F	0.99
nad5	1588	CTT	TTT	L->F	0.651
nad5	1607	CCC	CTC	P->L	0.984
nad5	1925	TCT	TTT	S->F	0.999
nad5	1927	CGT	TGT	R->C	0.999
nad5	1967	TCG	TTG	S->L	0.981
nad5	2009	TCA	TTA	S->L	0.804
nad6	26	CCA	CTA	P->L	0.991
nad6	53	GCA	GTA	A->V	0.999
nad6	88	CCC	TCC	P->S	0.991
nad6	89	CCC	CTC	P->L	0.998
nad6	90	CCC	CCT	P->P	0.557
nad6	95	CCA	CTA	P->L	0.994
nad6	103	CGC	TGC	R->C	0.979
nad6	146	TCC	TTC	S->F	1
nad6	161	CCA	CTA	P->L	0.999
nad6	169	CAT	TAT	H->Y	1
nad6	191	TCA	TTA	S->L	0.999
nad6	446	TCC	TTC	S->F	0.999
nad6	447	TCC	TCT	S->S	0.588
nad6	456	TTC	TTT	F->F	0.999
nad6	463	CCT	TCT	P->S	0.996
nad7	45	TTC	TTT	F->F	0.994
nad7	77	TCA	TTA	S->L	0.991
nad7	83	TCA	TTA	S->L	1
nad7	197	TCT	TTT	S->F	0.994
nad7	221	ACG	ATG	T->M	0.999
nad7	241	CAT	TAT	H->Y	0.998
nad7	248	TCA	TTA	S->L	0.993
nad7	312	TTC	TTT	F->F	0.61
nad7	313	CGT	TGT	R->C	0.997
nad7	332	TCA	TTA	S->L	0.999
nad7	341	TCA	TTA	S->L	0.999
nad7	380	TCA	TTA	S->L	1
nad7	528	TCC	TCT	S->S	0.522
nad7	530	TCC	TTC	S->F	0.998
nad7	575	TCA	TTA	S->L	1
nad7	676	CCA	TCA	P->S	0.987
nad7	695	TCG	TTG	S->L	0.995
nad7	721	CAT	TAT	H->Y	1
nad7	731	TCG	TTG	S->L	1
nad7	736	CCT	TCT	P->S	0.999
nad7	737	CCT	CTT	P->L	0.999

nad7	766	CGC	TGC	R->C	0.998
nad7	786	ATC	ATT	I->I	0.922
nad7	792	ATC	ATT	I->I	0.634
nad7	923	TCA	TTA	S->L	0.998
nad7	960	TCC	TCT	S->S	0.806
nad7	1047	CCC	CCT	P->P	0.966
nad7	1054	CGT	TGT	R->C	0.998
nad7	1076	TCT	TTT	S->F	0.999
nad7	1085	TCA	TTA	S->L	0.996
nad7	1100	TCT	TTT	S->F	0.999
nad7	1121	CCA	CTA	P->L	1
nad7	1134	GTC	GTT	V->V	0.979
nad7	1163	TCT	TTT	S->F	1
nad9	92	TCT	TTT	S->F	0.996
nad9	167	TCG	TTG	S->L	0.998
nad9	190	CAT	TAT	H->Y	1
nad9	298	CCG	TCG	P->S	0.996
nad9	328	CGG	TGG	R->W	1
nad9	368	TCC	TTC	S->F	1
nad9	398	TCA	TTA	S->L	0.998
nad9	439	CTT	TTT	L->F	0.998
nad9	534	TTC	TTT	F->F	0.524
sdh4	29	TCG	TTG	S->L	1
sdh4	155	CCA	CTA	P->L	0.983
sdh4	171	CTC	CTT	L->L	0.912
sdh4	191	TCC	TTC	S->F	0.608
sdh4	192	TCC	TCT	S->S	0.596
sdh4	203	TCA	TTA	S->L	0.935
sdh4	259	CAT	TAT	H->Y	0.9
sdh4	348	CTC	CTT	L->L	0.679
cob	53	ACA	ATA	T->I	0.685
cob	286	CTC	TTC	L->F	0.999
cob	298	CAC	TAC	H->Y	0.999
cob	325	CAT	TAT	H->Y	1
cob	358	CGG	TGG	R->W	1
cob	414	TAC	TAT	Y->Y	0.699
cob	568	CAT	TAT	H->Y	0.997
cob	725	TCT	TTT	S->F	0.999
cob	853	CAT	TAT	H->Y	0.999
cob	908	CCA	CTA	P->L	0.998
cob	982	CAC	TAC	H->Y	0.992
cob	1084	CCT	TCT	P->S	0.998
cob	1089	TTC	TTT	F->F	0.601
cob	1098	TTC	TTT	F->F	0.579
cob	1124	CCG	CTG	P->L	0.998
cob	1160	ACG	ATG	T->M	0.673
cox1	41	ACA	ATA	T->I	0.99
cox1	50	CCG	CTG	P->L	0.992
cox1	54	GTC	GTT	V->V	0.957
cox1	281	TCT	TTT	S->F	0.998
cox1	293	TCT	TTT	S->F	1
cox1	482	TCA	TTA	S->L	0.996
cox1	491	TCT	TTT	S->F	1
cox1	554	TCC	TTC	S->F	1
cox1	590	TCA	TTA	S->L	1
cox1	629	CCA	CTA	P->L	0.999
cox1	707	TCT	TTT	S->F	0.999
cox1	754	CGG	TGG	R->W	0.997
cox1	785	CCC	CTC	P->L	0.998
cox1	800	TCC	TTC	S->F	0.996
cox1	907	CGG	TGG	R->W	0.999
cox1	1076	TCC	TTC	S->F	0.999
cox1	1118	CCG	CTG	P->L	0.996
cox1	1163	GCG	GTG	A->V	0.999
cox1	1225	CAC	TAC	H->Y	1
cox1	1313	CCG	CTG	P->L	0.994
cox1	1318	CTC	TTC	L->F	0.987
cox1	1441	CGT	TGT	R->C	0.999
cox1	1472	TCA	TTA	S->L	0.999
cox2	27	CTC	CTT	L->L	0.618
cox2	38	CCT	CTT	P->L	0.69
cox2	71	TCT	TTT	S->F	1
cox2	138	CTC	CTT	L->L	0.934
cox2	138	CTC	CTT	L->L	0.923
cox2	161	TCA	TTA	S->L	0.993
cox2	161	TCA	TTA	S->L	0.999
cox2	163	CGG	TGG	R->W	1
cox2	253	CGG	TGG	R->W	0.998

cox2	278	CCG	CTG	P->L	0.998
cox2	379	CGG	TGG	R->W	0.998
cox2	443	ACG	ATG	T->M	0.999
cox2	460	CTA	TTA	L->L	0.658
cox2	476	TCA	TTA	S->L	1
cox2	544	CCT	TCT	P->S	0.999
cox2	557	CCT	CTT	P->L	0.996
cox2	581	TCA	TTA	S->L	1
cox2	632	TCG	TTG	S->L	0.999
cox2	698	TCT	TTT	S->F	0.939
cox2	718	CCT	TCT	P->S	0.984
cox2	739	CGG	TGG	R->W	0.952
cox3	112	CCA	TCA	P->S	0.998
cox3	257	TCT	TTT	S->F	0.995
cox3	289	CTT	TTT	L->F	1
cox3	304	CGG	TGG	R->W	0.998
cox3	311	TCT	TTT	S->F	0.947
cox3	314	TCT	TTT	S->F	0.968
cox3	413	CCT	CTT	P->L	0.995
cox3	512	TCA	TTA	S->L	1
cox3	565	CCC	TCC	P->S	0.999
cox3	566	CCC	CTC	P->L	0.995
cox3	653	TCG	TTG	S->L	1
cox3	754	CGG	TGG	R->W	1
atp1	1415	TCA	TTA	S->L	0.994
atp1	1490	CCA	CTA	P->L	0.999
atp4	40	CTA	TTA	L->L	0.913
atp4	59	TCT	TTT	S->F	0.995
atp4	89	TCA	TTA	S->L	0.998
atp4	138	ATC	ATT	I->I	0.755
atp4	215	TCG	TTG	S->L	0.997
atp4	227	CCA	CTA	P->L	0.989
atp4	251	GCG	GTG	A->V	0.562
atp4	414	GCC	GCT	A->A	0.716
atp4	416	ACT	ATT	T->I	0.967
atp4	448	CTA	TTA	L->L	0.576
atp6	22	CCG	TCG	P->S	0.635
atp6	35	CCG	CTG	P->L	0.751
atp6	124	CCA	TCA	P->S	0.544
atp6	125	CCA	CTA	P->L	0.85
atp6	125	CCA	CTA	P->L	0.949
atp6	200	TCT	TTT	S->F	0.609
atp6	218	ACT	ATT	T->I	0.715
atp6	291	CCC	CCT	P->P	0.537
atp6	322	CCT	TCT	P->S	0.637
atp6	361	CCA	TCA	P->S	1
atp6	403	CTT	TTT	L->F	0.623
atp6	440	TCA	TTA	S->L	1
atp6	491	CCG	CTG	P->L	0.999
atp6	497	CCG	CTG	P->L	0.996
atp6	560	TCG	TTG	S->L	0.998
atp6	578	TCG	TTG	S->L	0.998
atp6	586	CGT	TGT	R->C	0.999
atp6	593	CCC	CTC	P->L	0.997
atp6	594	CCC	CCT	P->P	0.968
atp6	725	TCA	TTA	S->L	0.999
atp6	784	CCT	TCT	P->S	0.999
atp6	787	CAT	TAT	H->Y	1
atp6	809	TCA	TTA	S->L	0.999
atp8	30	TTC	TTT	F->F	0.965
atp8	47	TCA	TTA	S->L	0.996
atp8	58	CTC	TTC	L->F	0.998
atp8	76	CCC	TCC	P->S	0.997
atp8	77	CCC	CTC	P->L	0.662
atp8	432	GAC	GAT	D->D	0.583
atp8	452	CCA	CTA	P->L	1
atp9	20	TCA	TTA	S->L	0.999
atp9	50	TCA	TTA	S->L	1
atp9	81	GTC	GTT	V->V	0.993
atp9	102	TCC	TCT	S->S	0.601
atp9	191	CCA	CTA	P->L	0.998
atp9	205	CTT	TTT	L->F	0.987
atp9	223	CGA	TGA	R->STOP	0.997
matR	44	TCC	TTC	S->F	0.886
matR	55	CCC	TCC	P->S	0.689
matR	205	CCA	TCA	P->S	0.994
matR	248	TCC	TTC	S->F	0.89
matR	266	TCT	TTT	S->F	0.77

matR	425	TCG	TTG	S->L	0.989
matR	935	TCA	TTA	S->L	1
matR	1557	CCC	CCT	P->P	0.876
matR	1691	TCC	TTC	S->F	0.996
matR	1712	CCT	CTT	P->L	1
matR	1746	TAC	TAT	Y->Y	0.998
matR	1768	CAC	TAC	H->Y	0.995
matR	1856	TCA	TTA	S->L	0.965
matR	1920	TCC	TCT	S->S	0.881
mttb	11	TCG	TTG	S->L	0.985
mttb	22	CTA	TTA	L->L	0.814
mttb	44	TCC	TTC	S->F	0.609
mttb	49	CGG	TGG	R->W	0.983
mttb	60	ATC	ATT	I->I	0.731
mttb	96	TTC	TTT	F->F	0.74
mttb	97	CCG	TCG	P->S	1
mttb	113	TCT	TTT	S->F	0.982
mttb	115	CCA	TCA	P->S	0.77
mttb	116	CCA	CTA	P->L	0.969
mttb	173	TCA	TTA	S->L	1
mttb	185	TCC	TTC	S->F	0.998
mttb	187	CCG	TCG	P->S	0.999
mttb	228	TTC	TTT	F->F	0.779
mttb	247	CAT	TAT	H->Y	0.999
mttb	270	ATC	ATT	I->I	0.729
mttb	316	CAT	TAT	H->Y	0.994
mttb	329	TCT	TTT	S->F	0.971
mttb	331	CGC	TGC	R->C	0.997
mttb	338	TCC	TTC	S->F	0.896
mttb	339	TCC	TCT	S->S	0.808
mttb	358	CCT	TCT	P->S	0.948
mttb	359	CCT	CTT	P->L	0.998
mttb	361	CCC	TCC	P->S	0.99
mttb	364	CGG	TGG	R->W	0.995
mttb	392	CCA	CTA	P->L	0.999
mttb	422	TCG	TTG	S->L	0.884
mttb	426	CTC	CTT	L->L	0.775
mttb	457	CAT	TAT	H->Y	0.997
mttb	482	TCG	TTG	S->L	0.994
mttb	490	CCA	TCA	P->S	0.981
mttb	504	TCC	TCT	S->S	0.626
mttb	526	CGT	TGT	R->C	1
mttb	533	CCA	CTA	P->L	0.996
mttb	539	CCG	CTG	P->L	0.988
mttb	601	CTC	TTC	L->F	0.886
mttb	645	ATC	ATT	I->I	0.988
mttb	652	CCT	TCT	P->S	0.98
mttb	653	CCT	CTT	P->L	0.687
mttb	665	TCT	TTT	S->F	0.767
mttb	698	TCG	TTG	S->L	0.999
ccmB	16	CTT	TTT	L->F	0.874
ccmB	28	CAT	TAT	H->Y	0.945
ccmB	39	ATC	ATT	I->I	0.665
ccmB	43	CCC	TCC	P->S	0.995
ccmB	70	CCG	TCG	P->S	0.905
ccmB	71	CCG	CTG	P->L	0.922
ccmB	80	TCG	TTG	S->L	0.975
ccmB	87	ATC	ATT	I->I	0.962
ccmB	137	TCC	TTC	S->F	0.997
ccmB	148	CCG	TCG	P->S	0.99
ccmB	149	CCG	CTG	P->L	0.992
ccmB	160	CCT	TCT	P->S	0.997
ccmB	164	CCG	CTG	P->L	0.995
ccmB	172	CCT	TCT	P->S	0.989
ccmB	179	CCT	CTT	P->L	0.988
ccmB	181	CCC	TCC	P->S	0.996
ccmB	183	CCC	CCT	P->P	0.542
ccmB	193	CCT	TCT	P->S	0.954
ccmB	194	CCT	CTT	P->L	0.997
ccmB	222	CTC	CTT	L->L	0.54
ccmB	286	CGG	TGG	R->W	0.999
ccmB	304	CGT	TGT	R->C	0.996
ccmB	337	CTG	TTG	L->L	0.569
ccmB	367	CGG	TGG	R->W	0.982
ccmB	379	CTA	TTA	L->L	0.938
ccmB	392	CCG	CTG	P->L	1
ccmB	406	CTG	TTG	L->L	0.749
ccmB	424	CGT	TGT	R->C	0.994

ccmB	428	TCG	TTG	S->L	0.996
ccmB	467	TCG	TTG	S->L	0.975
ccmB	475	CCA	TCA	P->S	0.997
ccmB	476	CCA	CTA	P->L	0.996
ccmB	485	TCA	TTA	S->L	0.985
ccmB	494	TCA	TTA	S->L	0.993
ccmB	503	TCA	TTA	S->L	0.973
ccmB	512	TCT	TTT	S->F	0.972
ccmB	514	CGT	TGT	R->C	0.997
ccmB	551	TCA	TTA	S->L	0.997
ccmB	554	TCG	TTG	S->L	0.994
ccmB	566	TCC	TTC	S->F	0.997
ccmB	569	TCT	TTT	S->F	0.979
ccmB	572	CCG	CTG	P->L	0.999
ccmB	576	TTC	TTT	F->F	0.566
ccmB	596	TCG	TTG	S->L	0.995
ccmB	611	TCA	TTA	S->L	0.835
ccmC	18	TTC	TTT	F->F	0.857
ccmC	45	ACC	ACT	T->T	0.535
ccmC	76	CGG	TGG	R->W	1
ccmC	84	TTC	TTT	F->F	0.532
ccmC	103	CAT	TAT	H->Y	0.999
ccmC	115	CGG	TGG	R->W	0.991
ccmC	133	CTT	TTT	L->F	0.987
ccmC	161	CCG	CTG	P->L	1
ccmC	179	GCG	GTG	A->V	0.999
ccmC	184	CGG	TGG	R->W	0.994
ccmC	331	CGG	TGG	R->W	0.999
ccmC	351	GGC	GGT	G->G	0.557
ccmC	395	TCG	TTG	S->L	0.995
ccmC	399	TTC	TTT	F->F	0.997
ccmC	400	CTT	TTT	L->F	0.997
ccmC	418	CTG	TTG	L->L	0.611
ccmC	421	CGT	TGT	R->C	0.987
ccmC	436	CCT	TCT	P->S	0.999
ccmC	446	CCG	CTG	P->L	0.996
ccmC	458	TCA	TTA	S->L	0.979
ccmC	463	CGT	TGT	R->C	0.999
ccmC	467	GCT	GTT	A->V	0.997
ccmC	473	CCG	CTG	P->L	0.995
ccmC	477	ATC	ATT	I->I	0.612
ccmC	497	TCT	TTT	S->F	0.999
ccmC	521	TCG	TTG	S->L	0.999
ccmC	548	TCT	TTT	S->F	0.96
ccmC	568	CCT	TCT	P->S	0.819
ccmC	568	CCT	TCT	P->S	0.686
ccmC	575	CCC	CTC	P->L	0.998
ccmC	606	TTC	TTT	F->F	0.514
ccmC	608	CCC	CTC	P->L	0.954
ccmC	614	TCA	TTA	S->L	0.982
ccmC	619	CGT	TGT	R->C	0.695
ccmC	624	ATC	ATT	I->I	0.888
ccmC	630	TTC	TTT	F->F	0.866
ccmC	650	CCT	CTT	P->L	0.993
ccmC	665	CCC	CTC	P->L	0.998
ccmFc	77	TCC	TTC	S->F	0.999
ccmFc	78	TCC	TCT	S->S	0.899
ccmFc	89	CCT	CTT	P->L	1
ccmFc	142	CCC	TCC	P->S	0.999
ccmFc	158	TCT	TTT	S->F	0.924
ccmFc	161	TCC	TTC	S->F	0.994
ccmFc	185	CCT	CTT	P->L	0.998
ccmFc	190	CCT	TCT	P->S	0.993
ccmFc	194	TCA	TTA	S->L	0.941
ccmFc	199	CCT	TCT	P->S	0.776
ccmFc	349	CGT	TGT	R->C	0.82
ccmFc	354	TTC	TTT	F->F	0.761
ccmFc	360	CCC	CCT	P->P	0.776
ccmFc	372	TTC	TTT	F->F	0.692
ccmFc	373	CTT	TTT	L->F	0.938
ccmFc	430	CGT	TGT	R->C	0.999
ccmFc	445	CGT	TGT	R->C	0.998
ccmFc	1052	TCT	TTT	S->F	0.99
ccmFc	1313	CCA	CTA	P->L	0.985
ccmFc	1334	TCG	TTG	S->L	0.999
ccmFc	1408	CGG	TGG	R->W	0.989
ccmFc	1433	TCG	TTG	S->L	0.989
ccmFc	1442	TCG	TTG	S->L	0.983

ccmFc	1489	CGA	TGA	R->STOP	0.994
ccmFn	112	CGG	TGG	R->W	0.585
ccmFn	196	CGG	TGG	R->W	0.91
ccmFn	278	CCG	CTG	P->L	0.999
ccmFn	338	CCT	CTT	P->L	1
ccmFn	377	TCG	TTG	S->L	0.762
ccmFn	382	CGT	TGT	R->C	0.939
ccmFn	391	CCG	TCG	P->S	0.773
ccmFn	392	CCG	CTG	P->L	0.815
ccmFn	488	TCA	TTA	S->L	0.999
ccmFn	496	CGG	TGG	R->W	0.999
ccmFn	503	CCA	CTA	P->L	1
ccmFn	519	TTC	TTT	F->F	0.672
ccmFn	523	CTT	TTT	L->F	0.993
ccmFn	612	TTC	TTT	F->F	0.917
ccmFn	640	CGT	TGT	R->C	0.52
ccmFn	947	CCT	CTT	P->L	0.999
ccmFn	956	TCA	TTA	S->L	0.986
ccmFn	994	CGT	TGT	R->C	0.998
ccmFn	999	ACC	ACT	T->T	0.852
ccmFn	1016	TCA	TTA	S->L	0.981
ccmFn	1028	CCA	CTA	P->L	0.999
ccmFn	1043	TCA	TTA	S->L	0.991
ccmFn	1192	CGC	TGC	R->C	0.957
ccmFn	1308	CTC	CTT	L->L	0.536
ccmFn	1427	TCG	TTG	S->L	0.886
ccmFn	1510	CGG	TGG	R->W	0.996
ccmFn	1538	CCA	CTA	P->L	1
ccmFn	1555	CAT	TAT	H->Y	1
ccmFn	1570	CGG	TGG	R->W	0.999
ccmFn	1621	CGG	TGG	R->W	0.999
ccmFn	1639	CGT	TGT	R->C	0.996
ccmFn	1682	TCG	TTG	S->L	0.957
ccmFn	1702	CTT	TTT	L->F	1
ccmFn	1809	TTC	TTT	F->F	0.602
rpl2	77	TCA	TTA	S->L	0.529
rpl2	215	CCA	CTA	P->L	0.995
rpl2	1200	TCC	TCT	S->S	0.597
rpl5	35	TCA	TTA	S->L	0.996
rpl5	59	CCG	CTG	P->L	0.969
rpl5	64	CAC	TAC	H->Y	0.912
rpl5	172	CTG	TTG	L->L	0.975
rpl5	175	CGC	TGC	R->C	1
rpl5	323	TCG	TTG	S->L	0.999
rpl5	335	TCG	TTG	S->L	0.946
rpl5	450	ATC	ATT	I->I	0.532
rpl5	518	CCA	CTA	P->L	0.97
rpl5	521	CCG	CTG	P->L	0.999
rpl10	9	TTC	TTT	F->F	0.576
rpl10	83	TCA	TTA	S->L	0.894
rpl10	101	TCG	TTG	S->L	0.995
rpl10	133	CTA	TTA	L->L	0.588
rpl10	239	TCG	TTG	S->L	0.996
rpl10	314	TCA	TTA	S->L	0.979
rpl10	330	TAC	TAT	Y->Y	0.804
rpl10	374	TCT	TTT	S->F	0.965
rpl16	102	GGC	GGT	G->G	0.814
rpl16	104	ACT	ATT	T->I	1
rpl16	164	ACA	ATA	T->I	1
rpl16	232	CTC	TTC	L->F	0.957
rpl16	321	TCC	TCT	S->S	0.913
rpl16	401	CCA	CTA	P->L	0.997
rpl16	407	TCG	TTG	S->L	0.992
rps1	26	TCA	TTA	S->L	0.678
rps1	45	TCC	TCT	S->S	0.86
rps1	161	CCT	CTT	P->L	0.96
rps1	212	TCC	TTC	S->F	0.867
rps1	629	TCG	TTG	S->L	0.878
rps3	69	TTC	TTT	F->F	0.974
rps3	69	TTC	TTT	F->F	0.974
rps3	92	TCA	TTA	S->L	0.997
rps3	92	TCA	TTA	S->L	0.997
rps3	96	GTC	GTT	V->V	0.578
rps3	96	GTC	GTT	V->V	0.578
rps3	126	TTC	TTT	F->F	0.574
rps3	126	TTC	TTT	F->F	0.574
rps3	512	TCA	TTA	S->L	0.992
rps3	512	TCA	TTA	S->L	0.992

rps3	699	CCC	CCT	P->P	0.802
rps3	699	CCC	CCT	P->P	0.802
rps3	713	TCG	TTG	S->L	0.993
rps3	713	TCG	TTG	S->L	0.993
rps3	763	CTC	TTC	L->F	0.619
rps3	763	CTC	TTC	L->F	0.619
rps3	890	TCG	TTG	S->L	0.905
rps3	890	TCG	TTG	S->L	0.905
rps3	1031	CCC	CTC	P->L	0.889
rps3	1031	CCC	CTC	P->L	0.889
rps3	1364	CCG	CTG	P->L	1
rps3	1364	CCG	CTG	P->L	1
rps3	1391	CCG	CTG	P->L	0.999
rps3	1391	CCG	CTG	P->L	0.999
rps3	1588	CCT	TCT	P->S	0.992
rps3	1588	CCT	TCT	P->S	0.992
rps4	38	TCA	TTA	S->L	0.932
rps4	38	TCA	TTA	S->L	0.932
rps4	49	CGG	TGG	R->W	1
rps4	49	CGG	TGG	R->W	1
rps4	164	TCA	TTA	S->L	0.999
rps4	164	TCA	TTA	S->L	0.999
rps4	193	CAT	TAT	H->Y	0.988
rps4	193	CAT	TAT	H->Y	0.988
rps4	266	CCA	CTA	P->L	0.99
rps4	266	CCA	CTA	P->L	0.99
rps4	278	TCG	TTG	S->L	0.997
rps4	278	TCG	TTG	S->L	0.997
rps4	290	CCG	CTG	P->L	0.999
rps4	290	CCG	CTG	P->L	0.999
rps4	307	CGT	TGT	R->C	0.984
rps4	307	CGT	TGT	R->C	0.984
rps4	335	CCG	CTG	P->L	0.996
rps4	335	CCG	CTG	P->L	0.996
rps4	474	ATC	ATT	I->I	0.673
rps4	474	ATC	ATT	I->I	0.673
rps4	482	TCA	TTA	S->L	0.983
rps4	482	TCA	TTA	S->L	0.983
rps4	668	TCA	TTA	S->L	0.662
rps4	668	TCA	TTA	S->L	0.662
rps4	693	TCC	TCT	S->S	0.685
rps4	693	TCC	TCT	S->S	0.685
rps4	840	ACC	ACT	T->T	0.652
rps4	840	ACC	ACT	T->T	0.652
rps4	885	ATC	ATT	I->I	0.944
rps4	885	ATC	ATT	I->I	0.944
rps4	894	ATC	ATT	I->I	0.786
rps4	894	ATC	ATT	I->I	0.786
rps4	914	TCG	TTG	S->L	0.995
rps4	914	TCG	TTG	S->L	0.995
rps4	925	CAT	TAT	H->Y	0.985
rps4	925	CAT	TAT	H->Y	0.985
rps4	950	TCT	TTT	S->F	0.997
rps4	950	TCT	TTT	S->F	0.997
rps4	1001	CCA	CTA	P->L	0.991
rps4	1001	CCA	CTA	P->L	0.991
rps4	1010	CCT	CTT	P->L	0.996
rps4	1010	CCT	CTT	P->L	0.996
rps4	1015	CGG	TGG	R->W	0.995
rps4	1015	CGG	TGG	R->W	0.995
rps7	177	CCC	CCT	P->P	0.619
rps7	177	CCC	CCT	P->P	0.619
rps7	332	TCA	TTA	S->L	0.986
rps7	332	TCA	TTA	S->L	0.986
rps10	62	ACG	ATG	T->M	0.998
rps10	298	CGG	TGG	R->W	0.986
rps10	310	CGT	TGT	R->C	0.633
rps10	358	CGG	TGG	R->W	0.995
rps10	387	TTC	TTT	F->F	0.962
rps10	398	TCA	TTA	S->L	0.643
rps12	284	TCC	TTC	S->F	0.999
rps13	5	TCA	TTA	S->L	0.518
rps13	26	TCA	TTA	S->L	0.999
rps13	56	TCA	TTA	S->L	0.995
rps13	100	CGT	TGT	R->C	0.964
rps13	193	CTT	TTT	L->F	0.767
rps13	287	TCG	TTG	S->L	0.999
rps14	194	TCC	TTC	S->F	0.932

rps14	271	CCT	TCT	P->S	0.744
rps19	138	TCC	TCT	S->S	0.977
rps19	163	CCT	TCT	P->S	0.958
rps19	164	CCT	CTT	P->L	0.947
rps19	180	ATC	ATT	I->I	0.777
psaB	40	CAG	TAG	Q->STOP	0.87
psaB	81	ACC	ACT	T->T	0.569
psaB	130	CAG	TAG	Q->STOP	0.98
psaB	175	CTG	TTG	L->L	0.784
psaB	185	TCC	TTC	S->F	0.625
psaB	353	TCT	TTT	S->F	0.535
psaB	410	ACA	ATA	T->I	0.591
psaB	433	CTT	TTT	L->F	0.953
psaB	504	TTC	TTT	F->F	0.596
psaB	525	CTC	CTT	L->L	0.67
psaB	539	TCG	TTG	S->L	0.741
psaB	549	TTC	TTT	F->F	0.843
psaB	599	CCT	CTT	P->L	0.504
psaB	649	CCG	TCG	P->S	0.795
psaB	655	CCC	TCC	P->S	0.69
psaB	680	ACA	ATA	T->I	0.586
psaB	710	CCC	CTC	P->L	0.602
psaB	716	TCA	TTA	S->L	0.9
psaB	740	TCC	TTC	S->F	0.592
psaB	774	CTC	CTT	L->L	0.72
psaB	823	CAT	TAT	H->Y	0.681
psaB	853	CTT	TTT	L->F	0.73
psaB	853	CTT	TTT	L->F	0.846
psaB	885	TTC	TTT	F->F	0.517
psaB	929	CCT	CTT	P->L	0.726
psaB	978	ATC	ATT	I->I	0.727
psaB	991	CAC	TAC	H->Y	0.959
psaB	997	CAA	TAA	Q->STOP	0.681
psaB	1012	CTA	TTA	L->L	0.839
psaB	1019	TCT	TTT	S->F	0.766
psaB	1165	CAT	TAT	H->Y	0.547
psaB	1182	TTC	TTT	F->F	0.628
psaB	1326	GTC	GTT	V->V	0.961
psaB	1381	CAA	TAA	Q->STOP	0.777
psaB	1399	CAT	TAT	H->Y	0.849
psaB	1485	CCC	CCT	P->P	0.672
psaB	1541	CCT	CTT	P->L	0.615
psaB	1622	GCA	GTA	A->V	0.633
psaB	1631	TCA	TTA	S->L	0.915
psaB	1659	TTC	TTT	F->F	0.858
psaB	1743	TTC	TTT	F->F	0.826
psaB	1797	ATC	ATT	I->I	0.673
psaB	1820	TCA	TTA	S->L	0.876
psaB	1838	TCC	TTC	S->F	0.778
psaB	1886	TCA	TTA	S->L	0.771
psaB	2102	TCC	TTC	S->F	0.853
psaB	2103	TCC	TCT	S->S	0.92
psaB	2134	CAC	TAC	H->Y	0.994
psaB	2141	TCT	TTT	S->F	0.882
psaB	2189	TCA	TTA	S->L	0.978
psbZ	11	TCC	TTC	S->F	0.625
psbZ	13	CTC	TTC	L->F	0.776
psbZ	158	TCA	TTA	S->L	0.764
psbZ	161	TCC	TTC	S->F	0.775
psbZ	182	CCC	CTC	P->L	0.931
psbZ	183	CCC	CCT	P->P	0.636
psbZ	200	CCT	CTT	P->L	0.588
psbZ	237	TCC	TCT	S->S	0.712
psbZ	280	CTT	TTT	L->F	0.694
psbZ	287	TCT	TTT	S->F	0.55
atpE	13	CTT	TTT	L->F	0.665
atpE	29	CCT	CTT	P->L	0.967
atpE	29	CCT	CTT	P->L	0.816
atpE	104	CCA	CTA	P->L	0.604
atpE	109	CAC	TAC	H->Y	0.706
atpE	116	CCT	CTT	P->L	0.571
atpE	166	CAA	TAA	Q->STOP	0.981
atpE	184	CTG	TTG	L->L	0.986
atpE	355	CTC	TTC	L->F	0.57
atpE	398	TCA	TTA	S->L	0.64
atpF	14	ACC	ATC	T->I	0.597
atpF	20	TCT	TTT	S->F	0.841
atpF	92	CCA	CTA	P->L	0.993

atpF	417	GTC	GTT	V->V	0.755
atpI	14	TCA	TTA	S->L	0.838
atpI	20	TCC	TTC	S->F	0.781
atpI	106	CAC	TAC	H->Y	0.689
atpI	158	TCA	TTA	S->L	0.677
atpI	194	CCA	CTA	P->L	0.603
atpI	205	CAG	TAG	Q->STOP	0.87
atpI	226	CTT	TTT	L->F	0.596
atpI	238	CGA	TGA	R->STOP	0.508
atpI	255	ACC	ACT	T->T	0.635
atpI	310	CTT	TTT	L->F	0.799
atpI	370	CAT	TAT	H->Y	0.856
atpI	370	CTA	TTA	L->L	0.944
atpI	483	TTC	TTT	F->F	0.594
atpI	492	TAC	TAT	Y->Y	0.617
atpI	540	TTC	TTT	F->F	0.589
atpI	610	CTT	TTT	L->F	0.758
atpI	617	TCT	TTT	S->F	0.808
atpI	637	CCT	TCT	P->S	0.878
atpI	637	CCT	TCT	P->S	0.709
atpI	638	CCT	CTT	P->L	0.585
atpI	638	CCT	CTT	P->L	0.886
atpI	644	CCT	CTT	P->L	0.609
atpI	644	CCT	CTT	P->L	0.945
atpI	685	CTT	TTT	L->F	0.62
petN	46	CCA	TCA	P->S	0.578
petN	59	TCA	TTA	S->L	0.533
petN	87	CTC	CTT	L->L	0.567
ndhC	30	TTC	TTT	F->F	0.827
ndhC	76	CTA	TTA	L->L	0.831
ndhC	148	CCA	TCA	P->S	0.553
ndhC	227	ACG	ATG	T->M	0.553
ndhC	241	CCA	TCA	P->S	0.505
ndhJ	113	TCC	TTC	S->F	0.829
ndhJ	182	CCC	CTC	P->L	0.812
ndhJ	183	CCC	CCT	P->P	0.682
ndhJ	292	CCG	TCG	P->S	0.583
ndhJ	293	CCG	CTG	P->L	0.788
ndhJ	447	CCC	CCT	P->P	0.61
ndhK	65	TCA	TTA	S->L	0.589
ndhK	95	TCT	TTT	S->F	0.778
ndhK	112	CTT	TTT	L->F	0.948
ndhK	135	TTC	TTT	F->F	0.765
ndhK	161	TCA	TTA	S->L	0.718
ndhK	205	CCT	TCT	P->S	0.605
ndhK	259	CCC	TCC	P->S	0.955
ndhK	345	TTC	TTT	F->F	0.62
ndhK	350	ACC	ATC	T->I	0.53
ndhK	351	ACC	ACT	T->T	0.692
ndhK	356	TCT	TTT	S->F	0.582
ndhK	392	CCT	CTT	P->L	0.503
ndhK	410	CCG	CTG	P->L	0.941
ndhK	493	CGA	TGA	R->STOP	0.6
ndhK	580	CAA	TAA	Q->STOP	0.634
ndhK	595	CAA	TAA	Q->STOP	0.871
ndhK	620	CCT	CTT	P->L	0.555
ndhK	622	CCT	TCT	P->S	0.68
ndhK	623	CCT	CTT	P->L	0.701
ndhK	661	CAC	TAC	H->Y	0.678
rps2	146	ACA	ATA	T->I	0,768
rps2	161	ACT	ATT	T->I	0,55
rps2	498	CCC	CCT	P->P	0,942
rps2	568	CCA	TCA	P->S	0,64
rps2	569	CCA	CTA	P->L	0,576
rps2	585	ATC	ATT	I->I	0,572
rps2	590	ACA	ATA	T->I	0,712
rps2	602	CCG	CTG	P->L	0,918
rps2	650	TCA	TTA	S->L	0,514
rps2	654	ATC	ATT	I->I	0,571
rps2	664	CTT	TTT	L->F	0,688
rps2	681	TTC	TTT	F->F	0,612
rps2	701	TCT	TTT	S->F	0,693
rps2	719	CCT	CTT	P->L	0,835
rps14-cp	61	CAT	TAT	H->Y	0.677
rps14-cp	70	CGT	TGT	R->C	0.821
rps14-cp	104	CCG	CTG	P->L	0.837
rps14-cp	151	CCG	TCG	P->S	0.842
rps14-cp	152	CCG	CTG	P->L	0.736

rps14-cp	167	CCT	CTT	P->L	0.691
rps14-cp	197	TCG	TTG	S->L	0.758
rps14-cp	250	CGG	TGG	R->W	0.761

Table S5: Mitochondrial RNA genes annotated in *Ludwigia grandiflora* subsp. *hexapetala* (*Lgh*) and *Ludwigia peploides* subsp. *montevidensis* (*Lpm*) mitogenomes. Locally Collinear Blocks (LCBs) correspond to conserved regions between *Lgh* and *Lpm* mitogenomes, identified by ProgressiveMauve.

Gene	LCB	Molecule
**tRNA**		
trnC-GCA	LCB0	M2
trnE-UUC	LCB5	M1
trnF-GAA	LCB4	M1
trnG-GCC	LCB1	M1
	LCB5	M1
trnH-GUG	LCB5	M1
trnK-UUU	LCB5	M1
trnM-CAU	LCB1	M1
	LCB4	M1
	LCB5	M1
	interLCB	M1
	interLCB	M1
	interLCB	M1
trnN-GUU	LCB0	M2
trnP-UGG	LCB4	M1
trnQ-UUG	LCB5	M1
trnS-GCU	LCB4	M1
trnS-UGA	LCB0	M2
trnY-GUA	LCB0	M2
**rRNA**		
rrn26	interLCB	M1
	interLCB	M1
	interLCB	M1
rrn5	LCB3	M1
	interLCB	M1
rrn18	LCB3	M1
	interLCB	M1

Table S6: Mobile elements in *Ludwigia grandiflora* subsp. *hexapetala* (A) and *Ludwigia peploides* subsp. *montevidensis* (B) mitogenomes. LINE stands for Long Interspersed Nuclear Elements, LTR refers to Long Terminal Repeat, and NERVE denotes Nonretroviral Endogenized RNA Virus Elements.

A

Ludwigia grandiflora* subsp. *hexapetala			
**Class**	**Coordinates**	**Length (bp)**	**Genes**
**Molecule M1**			
Class I / LINE	10,751-11,546	796	
	20,872-21,757	886	RH
	129,949-130,834	886	RH
	132,307-132,546	240	RH-like superfamily
	148,898-149,038	141	ENDO
	213,723-214,059	337	
	261,737-261,977	241	ENDO
	279,762-280,109	348	RH
	329,152-329,604	453	RT
	376,573-376,726	154	RT
	382,465-382,704	240	RH-like superfamily
	384,177-385,062	886	RH
	456,181-456,373	193	RT
	488,076-488,257	182	RT
	542,947-543,737	791	ENDO
Class I / LTR / Ty1-copia	3,928-4,132	205	PROT
	25,510-25,752	243	INT
	75,637-75,872	236	GAG
	148,595-148,792	198	RH
	176,581-176,676	96	RT
	353,782-353,973	192	RT
	404,483-405,581	1099	RT
Class I / LTR / Ty3-gypsy / chromovirus	42,455-42,728	274	INT
	166,953-168,071	1119	RH, RT
	183,238-183,359	122	GAG
	243,942-244,270	329	CHD, INT
	248,614-250,320	1707	PROT, INT, RT, RH
	265,861-266,763	903	RT, RH
	285,481-285,699	219	PROT
	429,875-430,905	1031	Integrase_H2C2 pfam17921 Integrase zinc binding domain
	479,719-479,991	273	INT
	492,676-496,668	3993	ENDO, RT, RH
	521,573-522,691	1119	RH, RT
Class I / LTR / Ty3-gypsy / non-chromovirus	53,135-54,241	1107	INT, RT
	88,483-89,405	923	RH, aRH
	166,168-166,592	425	RH
	320,597-320,828	232	PROT
	325,676-325,993	318	RNA-directed DNA polymerase
	397,214-397,401	188	RT
	457,374-457,550	177	RT
	457,707-457,853	147	RH
	523,052-523,476	425	RH
NERVE mitovirus	25,797-26,171	375	RNA-dependent RNA polymerase
	62,933-64,192	1260	RNA-directed DNA polymerase
	68,212-68,934	723	RNA-directed DNA polymerase
	150,031-150,348	318	RH domain
	168,815-169,924	1110	RNA-dependent RNA polymerase
	204,099-205,205	1107	RNA-dependent RNA polymerase, pfam05919
	426,804-427,376	573	RNA-dependent RNA polymerase
	446,229-446,951	723	RNA-directed DNA polymerase
	450,971-453,182	2212	RNA-directed DNA polymerase
	473,210-474,128	919	
	519,720-520,829	1110	RNA-dependent RNA polymerase
Plasmid-derived	291,293-294,250	2958	DNA polymerase type B
**Molecule M2**			
Class I / LINE	83,895-84,162	268	RT
	101,506-101,677	172	RT
	103,198-103,410	213	RH
Class I / LTR / Ty1-copia	16,980-17,060	81	RT
	21,095-21,382	288	GAG
	21,896-22,099	204	PROT
	22,112-22,696	585	INT
	23,502-24,269	768	RT
	24,312-24,407	96	RH
	24,538-24,919	382	RH
	64,264-64,356	93	RT
	88,990-89,664	675	RT
	157,481-157,821	341	RT
Class I / LTR / Ty3-gypsy / chromovirus	122,504-122,781	278	INT
Class I / LTR / Ty3-gypsy / non-chromovirus	91,156-91,488	333	RH
	133,044-133,419	376	INT
	135,340-135,889	550	INT
	150,771-150,887	117	aRH
	166,472-166,643	172	GAG
NERVE mitovirus	75,427-77,316	1890	RNA-dependent RNA polymerase

B

Ludwigia peploides subsp. *montevidensis*			
**Class**	**Coordinates**	**Length (bp)**	**Genes**
**Molecule M1**			
Class I / LINE	11,250-12,135	886	RH
	13,608-13,847	240	RH-like superfamily
	19,581-19,734	154	RT
	66,699-67,139	441	ENDO
	115,874-116,220	347	RH
	133,999-134,239	241	ENDO
	181,894-182,230	337	RT
	246,839-246,979	141	ENDO
	263,310-263,549	240	RH-like superfamily
	265,022-265,907	886	RH
	322,717-323,538	822	RT
	395,139-396,024	886	RH
	466,969-467,161	193	RT
	498,852-499,033	182	RT
	553,680-554,329	650	ENDO
Class I / LTR / Ty1-copia	3,927-4,131	205	PROT
	10,273-10,365	93	GAG
	42,318-42,509	192	RT
	219,222-219,317	96	RT
	247,085-247,281	197	RH
	266,792-266,884	93	GAG
	286,067-287,165	1099	RT
	341,637-341,739	103	INT
	391,146-391,387	242	INT
	396,909-397,001	93	GAG
	450,093-450,328	236	GAG
Class I / LTR / Ty3-gypsy / chromovirus	99,166-99,384	219	PROT
	129,206-130,114	909	RH, RT
	145,613-147,356	1744	INT, RH, RT, PROT
	151,705-152,032	328	INT, CHD
	212,545-212,666	122	GAG
	227,810-228,943	1134	RH, RT
	311,444-312,349	906	INT
	328,680-328,832	153	INT
	331,981-332,085	105	RH
	374,181-374,454	274	INT
	490,497-490,769	273	INT
	503,451-507,445	3995	ENDO, RT, RH
	532,321-533,454	1134	RH, RT
Class I / LTR / Ty3-gypsy / non-chromovirus	70,282-70,602	321	RT
	75,428-75,659	232	PROT
	229,303-229,726	424	RH
	278,803-278,990	188	RT
	362,702-363,794	1093	RT, INT
	436,549-437,504	956	aRH, RH
	468,162-468,338	177	RT
	468,495-468,641	147	RH
	533,814-534,237	424	RH
NERVE mitovirus	190,740-191,842	1103	RNA-dependent RNA polymerase, pfam05919
	225,971-227,080	1110	RNA-dependent RNA polymerase
	245,529-245,846	318	RH domain
	308,373-308,945	573	RNA-dependent RNA polymerase
	348,014-348,736	723	RNA-directed DNA polymerase
	352,755-354,014	1260	RNA-directed DNA polymerase
	390,728-391,101	374	RNA-dependent RNA polymerase
	457,022-457,744	723	RNA-directed DNA polymerase
	461,763-463,045	1283	RNA-directed DNA polymerase
	463,335-464,077	743	
	483,993-484,911	919	
	530,482-531,591	1110	RNA-dependent RNA polymerase
Plasmid-derived	104,764-107,721	2958	DNA polymerase type B
**Molecule M2**			
Class I / LINE	83,859-84,126	268	RT
	101,461-101,628	168	RT
	103,151-103,363	213	RH
Class I / LTR / Ty1-copia	16,976-17,056	81	RT
	21,091-21,378	288	GAG
	21,892-22,095	204	PROT
	23,498-24,265	768	RT
	24,308-24,403	96	RH
	24,534-24,915	382	RH
	64,245-64,337	93	RT
	88,953-89,627	675	RT
	157,381-157,721	341	RT
Class I / LTR / Ty3-gypsy / chromovirus	122,430-122,707	278	INT
Class I / LTR / Ty3-gypsy / non-chromovirus	91,117-91,448	332	RH
	132,966-133,341	376	INT
	135,262-135,811	550	INT
	150,675-150,791	117	aRH
	166,677-166,848	172	GAG
NERVE mitovirus	75,404-77,293	1890	RNA-dependent RNA polymerase

Table S7: Non-tandem repeated sequences in *Ludwigia grandiflora* subsp. *hexapetala* and *Ludwigia peploides* subsp. *montevidensis* mitogenomes.

Ludwigia grandiflora subsp. *hexapetala*							
**Molecule 1**	**Start 1**	**End 1**	**Molecule 2**	**Start 2**	**End 2**	**Direction**	**Length (bp)**
**Repeats M1-M1**							
M1	11549	21999	M1	120626	131076	forward	10451
M1	122334	132673	M1	382338	392677	reverse	10340
M1	163643	173026	M1	516618	526001	reverse	9384
M1	13257	21999	M1	383935	392677	reverse	8743
M1	62933	69195	M1	445968	452230	reverse	6263
M1	312774	313287	M1	437121	437634	reverse	514
M1	284951	285361	M1	297198	297608	reverse	411
M1	77591	77990	M1	184532	184931	reverse	400
M1	5195	5432	M1	17278	17515	forward	238
M1	5195	5432	M1	126355	126592	forward	238
M1	5195	5432	M1	388419	388656	reverse	238
M1	223090	223278	M1	316968	317156	forward	189
M1	415088	415275	M1	534368	534555	forward	188
M1	6538	6718	M1	367876	368056	reverse	181
M1	78043	78172	M1	184348	184477	reverse	130
M1	6577	6694	M1	438458	438575	forward	118
M1	367900	368017	M1	438458	438575	reverse	118
M1	367794	367910	M1	438585	438701	reverse	117
M1	7920	8032	M1	373826	373938	forward	113
M1	32962	33070	M1	148031	148139	forward	109
M1	145877	145972	M1	437035	437130	reverse	96
M1	194531	194625	M1	214479	214573	reverse	95
M1	108334	108418	M1	376774	376858	forward	85
M1	208207	208291	M1	368236	368320	forward	85
M1	34355	34432	M1	132192	132269	forward	78
M1	34355	34432	M1	382742	382819	reverse	78
M1	288585	288662	M1	333946	334023	forward	78
M1	120301	120374	M1	176144	176217	reverse	74
M1	78216	78288	M1	184232	184304	reverse	73
M1	19798	19860	M1	355714	355776	reverse	63
M1	69520	69582	M1	276915	276977	forward	63
M1	128875	128937	M1	355714	355776	reverse	63
M1	355714	355776	M1	386074	386136	forward	63
M1	143419	143480	M1	309849	309910	forward	62
M1	16901	16961	M1	338343	338403	forward	61
M1	125978	126038	M1	338343	338403	forward	61
M1	167874	167934	M1	249770	249830	forward	61
M1	249770	249830	M1	521710	521770	reverse	61
M1	338343	338403	M1	388973	389033	reverse	61
M1	31789	31848	M1	526931	526990	reverse	60
M1	44814	44873	M1	276543	276602	reverse	60
M1	528899	528957	M1	538059	538117	forward	59
M1	2423	2479	M1	38260	38316	forward	57
M1	31850	31906	M1	526873	526929	reverse	57
M1	194413	194469	M1	214612	214668	reverse	57
M1	6513	6568	M1	438394	438449	forward	56
M1	2373	2427	M1	38205	38259	forward	55
M1	49272	49325	M1	263886	263939	reverse	54
M1	77995	78047	M1	184475	184527	reverse	53
M1	82334	82385	M1	164604	164655	reverse	52
M1	82334	82385	M1	524989	525040	forward	52
M1	34434	34484	M1	132271	132321	forward	51
M1	34434	34484	M1	382690	382740	reverse	51
M1	288229	288279	M1	291066	291116	forward	51
M1	74955	75004	M1	236571	236620	reverse	50
M1	17913	17960	M1	255010	255057	forward	48
M1	126990	127037	M1	255010	255057	forward	48
M1	255010	255057	M1	387974	388021	reverse	48
M1	16817	16863	M1	337854	337900	forward	47
M1	54633	54679	M1	369262	369308	forward	47
M1	125894	125940	M1	337854	337900	forward	47
M1	321812	321858	M1	381274	381320	forward	47
M1	321936	321982	M1	381401	381447	forward	47
M1	337854	337900	M1	389071	389117	reverse	47

M1	288591	288636	M1	368106	368151	forward	46
M1	333952	333997	M1	368106	368151	forward	46
M1	225383	225426	M1	456557	456600	forward	44
M1	337253	337296	M1	490941	490984	reverse	44
M1	45486	45528	M1	433404	433446	reverse	43
M1	145210	145252	M1	289836	289878	forward	43
M1	151915	151957	M1	317898	317940	reverse	43
M1	370264	370306	M1	431518	431560	forward	43
M1	475310	475352	M1	516536	516578	reverse	43
M1	221	262	M1	378372	378413	reverse	42
M1	20065	20106	M1	142258	142299	reverse	42
M1	129142	129183	M1	142258	142299	reverse	42
M1	142258	142299	M1	385828	385869	forward	42
M1	34265	34305	M1	67335	67375	forward	41
M1	34265	34305	M1	447788	447828	reverse	41
M1	20002	20041	M1	509995	510034	reverse	40
M1	129079	129118	M1	509995	510034	reverse	40
M1	133896	133935	M1	284645	284684	reverse	40
M1	288599	288638	M1	438373	438412	reverse	40
M1	333960	333999	M1	438373	438412	reverse	40
M1	385893	385932	M1	509995	510034	forward	40
M1	34666	34704	M1	459040	459078	reverse	39
M1	45452	45490	M1	433449	433487	reverse	39
M1	78783	78821	M1	509998	510036	forward	39
M1	90452	90490	M1	236609	236647	forward	39
M1	149182	149220	M1	152789	152827	forward	39
M1	394376	394414	M1	470990	471028	reverse	39
M1	20388	20425	M1	315480	315517	forward	38
M1	91063	91100	M1	208160	208197	forward	38
M1	99975	100012	M1	198279	198316	forward	38
M1	108498	108535	M1	376938	376975	forward	38
M1	129465	129502	M1	315480	315517	forward	38
M1	228460	228497	M1	321182	321219	reverse	38
M1	315480	315517	M1	385509	385546	reverse	38
M1	368114	368151	M1	438375	438412	reverse	38
M1	20002	20038	M1	78783	78819	reverse	37
M1	55420	55456	M1	526995	527031	forward	37
M1	78783	78819	M1	129079	129115	reverse	37
M1	78783	78819	M1	385896	385932	forward	37
M1	178967	179003	M1	299580	299616	forward	37
M1	6413	6448	M1	192430	192465	forward	36
M1	144236	144271	M1	230033	230068	reverse	36
M1	378795	378830	M1	398734	398769	reverse	36
M1	6684	6718	M1	438585	438619	forward	35
M1	18481	18515	M1	66057	66091	forward	35
M1	18481	18515	M1	449072	449106	reverse	35
M1	66057	66091	M1	127558	127592	forward	35
M1	66057	66091	M1	387419	387453	reverse	35
M1	66977	67011	M1	105581	105615	reverse	35
M1	77684	77718	M1	189539	189573	reverse	35
M1	105581	105615	M1	448152	448186	forward	35
M1	127558	127592	M1	449072	449106	reverse	35
M1	184804	184838	M1	189539	189573	forward	35
M1	225465	225499	M1	500436	500470	forward	35
M1	387419	387453	M1	449072	449106	forward	35
M1	12568	12601	M1	392678	392711	forward	34
M1	121645	121678	M1	392678	392711	forward	34
M1	150385	150418	M1	194972	195005	reverse	34
M1	179010	179043	M1	299623	299656	forward	34
M1	316387	316420	M1	346497	346530	reverse	34
M1	321724	321757	M1	381193	381226	forward	34
M1	54744	54776	M1	369383	369415	forward	33
M1	66377	66409	M1	106191	106223	reverse	33
M1	79477	79509	M1	137937	137969	forward	33
M1	106191	106223	M1	448754	448786	forward	33
M1	142723	142755	M1	229919	229951	reverse	33
M1	171606	171638	M1	476145	476177	forward	33
M1	280769	280801	M1	372920	372952	forward	33
M1	321601	321633	M1	381081	381113	forward	33
M1	476145	476177	M1	518006	518038	reverse	33
M1	476146	476178	M1	497217	497249	forward	33
M1	45530	45561	M1	433371	433402	reverse	32
M1	79431	79462	M1	137892	137923	forward	32
M1	100222	100253	M1	203624	203655	forward	32
M1	170512	170543	M1	476102	476133	forward	32
M1	171607	171638	M1	497217	497248	forward	32
M1	312383	312414	M1	345838	345869	forward	32
M1	476102	476133	M1	519101	519132	reverse	32

M1	497217	497248	M1	518006	518037	reverse	32
M1	64993	65023	M1	183790	183820	forward	31
M1	170930	170960	M1	289925	289955	forward	31
M1	183790	183820	M1	450140	450170	reverse	31
M1	289925	289955	M1	518684	518714	reverse	31
M1	368026	368056	M1	438419	438449	reverse	31
M1	438208	438238	M1	438217	438247	forward	31
M1	20512	20541	M1	315558	315587	forward	30
M1	22928	22957	M1	170028	170057	forward	30
M1	22928	22957	M1	519587	519616	reverse	30
M1	75027	75056	M1	215550	215579	forward	30
M1	129589	129618	M1	315558	315587	forward	30
M1	171929	171958	M1	171970	171999	forward	30
M1	171929	171958	M1	517645	517674	reverse	30
M1	171970	171999	M1	517686	517715	reverse	30
M1	309773	309802	M1	415231	415260	reverse	30
M1	309773	309802	M1	534511	534540	reverse	30
M1	315558	315587	M1	385393	385422	reverse	30
M1	517645	517674	M1	517686	517715	forward	30

Repeats M1-M2

M1	414973	415421	M2	7384	7832	forward	449
M1	309847	310212	M2	4205	4570	reverse	366
M1	438375	438569	M2	140233	140427	reverse	195
M1	534368	534555	M2	7499	7686	forward	188
M1	260642	260825	M2	139747	139930	reverse	184
M1	6684	6849	M2	140064	140229	reverse	166
M1	131199	131351	M2	98976	99128	forward	153
M1	383660	383812	M2	98976	99128	reverse	153
M1	526048	526180	M2	70417	70549	forward	133
M1	6577	6688	M2	140233	140344	reverse	112
M1	367906	368017	M2	140233	140344	forward	112
M1	336061	336170	M2	75597	75706	forward	110
M1	526822	526929	M2	12068	12175	forward	108
M1	152756	152860	M2	77917	78021	reverse	105
M1	368114	368214	M2	140390	140490	forward	101
M1	31807	31906	M2	12119	12218	reverse	100
M1	101172	101269	M2	70284	70381	forward	98
M1	219940	220034	M2	95784	95878	reverse	95
M1	90954	91045	M2	80915	81006	reverse	92
M1	260827	260909	M2	139663	139745	reverse	83
M1	310262	310340	M2	2423	2501	reverse	79
M1	208120	208197	M2	80866	80943	reverse	78
M1	13643	13718	M2	98040	98115	forward	76
M1	122720	122795	M2	98040	98115	forward	76
M1	392216	392291	M2	98040	98115	reverse	76
M1	337531	337605	M2	143363	143437	reverse	75
M1	260569	260640	M2	139932	140003	reverse	72
M1	3552	3616	M2	104366	104430	forward	65
M1	193868	193932	M2	10275	10339	reverse	65
M1	143419	143480	M2	4507	4568	reverse	62
M1	300311	300372	M2	114078	114139	forward	62
M1	328849	328909	M2	11051	11111	forward	61
M1	193349	193407	M2	10545	10603	reverse	59
M1	277474	277532	M2	134536	134594	reverse	59
M1	183734	183791	M2	3186	3243	forward	58
M1	483474	483531	M2	38032	38089	forward	58
M1	6513	6568	M2	140353	140408	reverse	56
M1	114221	114276	M2	82507	82562	reverse	56
M1	250912	250966	M2	15374	15428	forward	55
M1	193470	193521	M2	10426	10477	reverse	52
M1	6851	6901	M2	140012	140062	reverse	51
M1	91063	91112	M2	80854	80903	reverse	50
M1	193813	193862	M2	10345	10394	reverse	50
M1	237530	237579	M2	6030	6079	forward	50
M1	237581	237629	M2	6081	6129	forward	49
M1	527845	527892	M2	28630	28677	reverse	48
M1	31836	31882	M2	11360	11406	reverse	47
M1	284529	284575	M2	74380	74426	forward	47
M1	10207	10252	M2	67926	67971	forward	46
M1	54422	54467	M2	41295	41340	reverse	46
M1	219380	219425	M2	87566	87611	reverse	46
M1	371703	371747	M2	156725	156769	reverse	45
M1	250867	250910	M2	15329	15372	forward	44
M1	277930	277973	M2	138175	138218	forward	44
M1	3668	3709	M2	103545	103586	reverse	42
M1	11767	11808	M2	67095	67136	reverse	42

M1	120844	120885	M2	67095	67136	reverse	42
M1	526931	526972	M2	12177	12218	forward	42
M1	149182	149220	M2	77950	77988	reverse	39
M1	334028	334066	M2	56171	56209	reverse	39
M1	495720	495758	M2	338	376	reverse	39
M1	11609	11646	M2	67265	67302	reverse	38
M1	120686	120723	M2	67265	67302	reverse	38
M1	288599	288636	M2	140390	140427	forward	38
M1	333960	333997	M2	140390	140427	forward	38
M1	525996	526033	M2	70366	70403	forward	38
M1	438583	438619	M2	140195	140231	reverse	37
M1	452906	452942	M2	75691	75727	reverse	37
M1	11655	11690	M2	67221	67256	reverse	36
M1	120732	120767	M2	67221	67256	reverse	36
M1	172165	172200	M2	153336	153371	forward	36
M1	398737	398772	M2	114244	114279	reverse	36
M1	517444	517479	M2	153336	153371	reverse	36
M1	312739	312773	M2	140538	140572	forward	35
M1	337489	337523	M2	143447	143481	reverse	35
M1	367876	367910	M2	140195	140229	forward	35
M1	505035	505068	M2	3638	3671	reverse	34
M1	527812	527845	M2	28676	28709	reverse	34
M1	17539	17571	M2	26713	26745	reverse	33
M1	126616	126648	M2	26713	26745	reverse	33
M1	180834	180866	M2	114667	114699	reverse	33
M1	228460	228492	M2	52856	52888	reverse	33
M1	321187	321219	M2	52856	52888	forward	33
M1	336028	336060	M2	75566	75598	forward	33
M1	378795	378827	M2	114247	114279	forward	33
M1	388363	388395	M2	26713	26745	forward	33
M1	526897	526929	M2	11360	11392	forward	33
M1	278160	278191	M2	10875	10906	reverse	32
M1	321189	321220	M2	33211	33242	reverse	32
M1	465213	465244	M2	61620	61651	forward	32
M1	542203	542234	M2	154385	154416	reverse	32
M1	228460	228490	M2	33212	33242	forward	31
M1	368026	368056	M2	140353	140383	forward	31
M1	378575	378605	M2	116632	116662	reverse	31
M1	35780	35809	M2	5418	5447	forward	30
M1	99593	99622	M2	84402	84431	reverse	30
M1	309773	309802	M2	7642	7671	reverse	30
M1	411878	411907	M2	111492	111521	forward	30

Repeats M2-M2

M2	70688	70753	M2	70817	70882	forward	66
M2	11411	11458	M2	12194	12241	forward	48
M2	54051	54098	M2	145529	145576	reverse	48
M2	11360	11406	M2	12143	12189	forward	47
M2	107670	107710	M2	142149	142189	reverse	41
M2	138121	138159	M2	151685	151723	reverse	39
M2	33212	33242	M2	52858	52888	reverse	31

Ludwigia peploides* subsp. *montevidensis

Repeats M1-M1

M1	264780	274180	M1	394897	404297	forward	9401
M1	222869	232244	M1	527380	536755	forward	9376
M1	346199	354003	M1	455207	463011	forward	7805
M1	6737	13908	M1	263249	270420	reverse	7172
M1	6737	12377	M1	394897	400537	reverse	5641
M1	210976	211556	M1	447793	448373	reverse	581
M1	82969	83482	M1	318686	319199	forward	514
M1	296661	296887	M1	545119	545345	forward	227
M1	6131	6324	M1	61904	62097	forward	194
M1	5902	6061	M1	61669	61828	forward	160
M1	79136	79292	M1	172716	172872	forward	157
M1	28282	28423	M1	320020	320161	forward	142
M1	146305	146413	M1	331979	332087	forward	109
M1	247735	247843	M1	383837	383945	forward	109
M1	6636	6738	M1	201799	201901	reverse	103
M1	249899	249994	M1	318600	318695	forward	96
M1	181382	181476	M1	201305	201399	reverse	95
M1	5799	5887	M1	61566	61654	forward	89
M1	19449	19533	M1	417565	417649	forward	85

M1	27979	28063	M1	187661	187745	forward	85
M1	13493	13570	M1	382475	382552	reverse	78
M1	263587	263664	M1	382475	382552	forward	78
M1	62258	62334	M1	102065	102141	reverse	77
M1	96580	96656	M1	216858	216934	forward	77
M1	219682	219755	M1	405594	405667	reverse	74
M1	13901	13973	M1	263185	263257	reverse	73
M1	211600	211672	M1	447677	447749	reverse	73
M1	329221	329291	M1	512329	512399	forward	71
M1	58980	59043	M1	501711	501774	forward	64
M1	10176	10238	M1	40517	40579	forward	63
M1	40517	40579	M1	266919	266981	reverse	63
M1	40517	40579	M1	397036	397098	reverse	63
M1	119004	119066	M1	347367	347429	forward	63
M1	119004	119066	M1	456375	456437	forward	63
M1	7279	7340	M1	57878	57939	reverse	62
M1	57878	57939	M1	269817	269878	forward	62
M1	57878	57939	M1	399934	399995	forward	62
M1	86336	86397	M1	252390	252451	forward	62
M1	146137	146197	M1	227961	228021	forward	61
M1	146137	146197	M1	532472	532532	forward	61
M1	385059	385118	M1	537681	537740	forward	60
M1	539648	539706	M1	548799	548857	forward	59
M1	2425	2481	M1	378590	378646	reverse	57
M1	181287	181343	M1	201461	201517	reverse	57
M1	385001	385057	M1	537624	537680	forward	57
M1	2375	2429	M1	378647	378701	reverse	55
M1	132038	132091	M1	367616	367669	reverse	54
M1	231236	231287	M1	443581	443632	reverse	52
M1	443581	443632	M1	535747	535798	reverse	52
M1	13572	13622	M1	382423	382473	reverse	51
M1	263535	263585	M1	382423	382473	forward	51
M1	146412	146461	M1	332087	332136	forward	50
M1	159351	159400	M1	450956	451005	reverse	50
M1	297661	297710	M1	359632	359681	forward	50
M1	14864	14910	M1	74281	74327	forward	47
M1	14991	15037	M1	74403	74449	forward	47
M1	17478	17524	M1	358924	358970	forward	47
M1	26993	27039	M1	362252	362298	forward	47
M1	339162	339208	M1	526061	526107	reverse	47
M1	28148	28193	M1	62283	62328	forward	46
M1	28148	28193	M1	102071	102116	reverse	46
M1	8291	8335	M1	140921	140965	reverse	45
M1	140921	140965	M1	268822	268866	forward	45
M1	140921	140965	M1	398939	398983	forward	45
M1	170538	170581	M1	467345	467388	reverse	44
M1	25995	26037	M1	313087	313129	reverse	43
M1	26920	26962	M1	362189	362231	forward	43
M1	78322	78364	M1	243920	243962	reverse	43
M1	103307	103349	M1	250619	250661	reverse	43
M1	314972	315014	M1	371393	371435	forward	43
M1	486092	486134	M1	527298	527340	reverse	43
M1	223	264	M1	17896	17937	forward	42
M1	7195	7236	M1	58387	58428	reverse	42
M1	10443	10484	M1	253567	253608	forward	42
M1	58387	58428	M1	269921	269962	forward	42
M1	58387	58428	M1	400038	400079	forward	42
M1	253567	253608	M1	266673	266714	reverse	42
M1	253567	253608	M1	396790	396831	reverse	42
M1	539708	539749	M1	548859	548900	forward	42
M1	28359	28399	M1	329751	329791	forward	41
M1	98556	98596	M1	111075	111115	reverse	41
M1	274262	274302	M1	404290	404330	reverse	41
M1	320097	320137	M1	329751	329791	forward	41
M1	333098	333138	M1	443444	443484	forward	41
M1	349573	349613	M1	382602	382642	forward	41
M1	382602	382642	M1	458581	458621	forward	41
M1	10380	10419	M1	520763	520802	reverse	40
M1	62281	62320	M1	319935	319974	forward	40
M1	101976	102015	M1	203291	203330	forward	40
M1	102079	102118	M1	319935	319974	reverse	40
M1	111302	111341	M1	261925	261964	reverse	40
M1	126852	126891	M1	169408	169447	forward	40
M1	266738	266777	M1	520763	520802	forward	40
M1	396855	396894	M1	520763	520802	forward	40
M1	104536	104574	M1	223833	223871	forward	39
M1	104536	104574	M1	528344	528382	forward	39
M1	159324	159362	M1	435477	435515	forward	39

M1	225829	225867	M1	393931	393969	forward	39
M1	243052	243090	M1	246657	246695	forward	39
M1	275967	276005	M1	481773	481811	reverse	39
M1	315017	315055	M1	371431	371469	forward	39
M1	334685	334723	M1	435476	435514	reverse	39
M1	382203	382241	M1	469828	469866	forward	39
M1	393931	393969	M1	530340	530378	forward	39
M1	447144	447182	M1	520766	520804	reverse	39
M1	10379	10416	M1	72778	72815	reverse	38
M1	10766	10803	M1	80740	80777	reverse	38
M1	19332	19369	M1	417448	417485	forward	38
M1	28148	28185	M1	319937	319974	forward	38
M1	72778	72815	M1	266741	266778	forward	38
M1	72778	72815	M1	396858	396895	forward	38
M1	75042	75079	M1	167467	167504	reverse	38
M1	80740	80777	M1	266354	266391	forward	38
M1	80740	80777	M1	396471	396508	forward	38
M1	159324	159361	M1	334685	334722	reverse	38
M1	180552	180589	M1	549889	549926	reverse	38
M1	187755	187792	M1	434869	434906	forward	38
M1	197619	197656	M1	425970	426007	forward	38
M1	10380	10416	M1	447146	447182	forward	37
M1	50463	50499	M1	351009	351045	reverse	37
M1	50463	50499	M1	460017	460053	reverse	37
M1	72778	72814	M1	447146	447182	reverse	37
M1	72778	72814	M1	520766	520802	forward	37
M1	266741	266777	M1	447146	447182	reverse	37
M1	361477	361513	M1	537745	537781	reverse	37
M1	396858	396894	M1	447146	447182	reverse	37
M1	17480	17515	M1	280322	280357	forward	36
M1	72776	72811	M1	358897	358932	forward	36
M1	165897	165932	M1	251600	251635	reverse	36
M1	280322	280357	M1	358926	358961	forward	36
M1	8859	8893	M1	350857	350891	reverse	35
M1	8859	8893	M1	459865	459899	reverse	35
M1	170465	170499	M1	511209	511243	reverse	35
M1	206354	206388	M1	211069	211103	forward	35
M1	206354	206388	M1	448246	448280	reverse	35
M1	268264	268298	M1	350857	350891	forward	35
M1	268264	268298	M1	459865	459899	forward	35
M1	319773	319807	M1	319782	319816	forward	35
M1	349937	349971	M1	420362	420396	reverse	35
M1	350857	350891	M1	398381	398415	forward	35
M1	398381	398415	M1	459865	459899	forward	35
M1	420362	420396	M1	458945	458979	reverse	35
M1	10383	10416	M1	358899	358932	reverse	34
M1	15085	15118	M1	74504	74537	forward	34
M1	49757	49790	M1	79839	79872	reverse	34
M1	200925	200958	M1	245459	245492	reverse	34
M1	266741	266774	M1	358899	358932	forward	34
M1	358899	358932	M1	396858	396891	forward	34
M1	358899	358932	M1	447149	447182	reverse	34
M1	358899	358932	M1	520766	520799	forward	34
M1	15198	15230	M1	74628	74660	forward	33
M1	23350	23382	M1	115183	115215	forward	33
M1	79105	79137	M1	172684	172716	forward	33
M1	110449	110481	M1	110506	110538	forward	33
M1	166014	166046	M1	253114	253146	reverse	33
M1	224257	224289	M1	486927	486959	reverse	33
M1	257894	257926	M1	446456	446488	forward	33
M1	350539	350571	M1	419754	419786	reverse	33
M1	419754	419786	M1	459547	459579	reverse	33
M1	486927	486959	M1	528768	528800	reverse	33
M1	486928	486960	M1	507990	508022	forward	33
M1	26887	26918	M1	362156	362187	forward	32
M1	50418	50449	M1	83840	83871	forward	32
M1	180376	180407	M1	450904	450935	forward	32
M1	192285	192316	M1	425729	425760	forward	32
M1	224257	224288	M1	507990	508021	reverse	32
M1	225352	225383	M1	486884	486915	reverse	32
M1	257940	257971	M1	446503	446534	forward	32
M1	486884	486915	M1	529863	529894	reverse	32
M1	507990	508021	M1	528768	528799	reverse	32
M1	5767	5797	M1	61534	61564	forward	31
M1	28243	28273	M1	319981	320011	forward	31
M1	103396	103426	M1	224935	224965	reverse	31
M1	103396	103426	M1	529446	529476	reverse	31
M1	212084	212114	M1	351924	351954	forward	31

M1	212084	212114	M1	460932	460962	forward	31
M1	10890	10919	M1	80670	80699	reverse	30
M1	80670	80699	M1	266238	266267	forward	30
M1	80670	80699	M1	396355	396384	forward	30
M1	86444	86473	M1	296804	296833	forward	30
M1	86444	86473	M1	545262	545291	forward	30
M1	223896	223925	M1	223937	223966	forward	30
M1	223896	223925	M1	528448	528477	forward	30
M1	223937	223966	M1	528407	528436	forward	30
M1	528407	528436	M1	528448	528477	forward	30

Repeats M1-M2

M1	296546	296994	M2	7381	7829	forward	449
M1	86034	86399	M2	4204	4569	forward	366
M1	319937	320186	M2	140087	140336	reverse	250
M1	545119	545345	M2	7496	7722	forward	227
M1	135150	135333	M2	139664	139847	forward	184
M1	12500	12652	M2	98932	99084	forward	153
M1	264505	264657	M2	98932	99084	reverse	153
M1	28282	28423	M2	140112	140253	reverse	142
M1	6473	6611	M2	11398	11536	reverse	139
M1	536802	536934	M2	70395	70527	forward	133
M1	60111	60220	M2	75574	75683	reverse	110
M1	537573	537680	M2	12065	12172	forward	108
M1	28085	28185	M2	140299	140399	reverse	101
M1	385001	385100	M2	12116	12215	forward	100
M1	424717	424814	M2	70262	70359	reverse	98
M1	320185	320281	M2	139993	140089	reverse	97
M1	175927	176021	M2	95742	95836	forward	95
M1	434924	435015	M2	80885	80976	forward	92
M1	135066	135148	M2	139580	139662	forward	83
M1	85906	85984	M2	2423	2501	forward	79
M1	187755	187832	M2	80836	80913	forward	78
M1	273060	273135	M2	97996	98071	reverse	76
M1	403177	403252	M2	97996	98071	reverse	76
M1	58676	58750	M2	143270	143344	forward	75
M1	135335	135406	M2	139849	139920	forward	72
M1	243052	243123	M2	77923	77994	forward	72
M1	3552	3616	M2	104319	104383	forward	65
M1	201996	202060	M2	10271	10335	forward	65
M1	95864	95925	M2	114015	114076	reverse	62
M1	252390	252451	M2	4506	4567	forward	62
M1	67367	67427	M2	11047	11107	reverse	61
M1	118449	118507	M2	134458	134516	forward	59
M1	202521	202579	M2	10541	10599	forward	59
M1	212113	212170	M2	3185	3242	reverse	58
M1	494253	494310	M2	38023	38080	forward	58
M1	328938	328994	M2	73650	73706	reverse	57
M1	411694	411749	M2	82473	82528	forward	56
M1	145001	145055	M2	15371	15425	reverse	55
M1	320294	320346	M2	139928	139980	reverse	53
M1	202407	202458	M2	10422	10473	forward	52
M1	158392	158441	M2	6029	6078	reverse	50
M1	202066	202115	M2	10341	10390	forward	50
M1	434857	434906	M2	80824	80873	forward	50
M1	6554	6601	M2	12191	12238	reverse	48
M1	538595	538642	M2	28625	28672	reverse	48
M1	111411	111457	M2	74357	74403	reverse	47
M1	385025	385071	M2	11357	11403	forward	47
M1	176536	176581	M2	87529	87574	forward	46
M1	362466	362511	M2	41283	41328	forward	46
M1	24554	24598	M2	156626	156670	forward	45
M1	118008	118051	M2	138095	138138	reverse	44
M1	145057	145100	M2	15326	15369	reverse	44
M1	463144	463187	M2	76217	76260	reverse	44
M1	3668	3709	M2	103498	103539	reverse	42
M1	6426	6467	M2	141272	141313	reverse	42
M1	405083	405124	M2	67074	67115	forward	42
M1	537681	537722	M2	12174	12215	forward	42
M1	246655	246695	M2	77921	77961	forward	41
M1	329751	329791	M2	140136	140176	reverse	41
M1	62214	62252	M2	56158	56196	forward	39
M1	506495	506533	M2	338	376	reverse	39
M1	62283	62320	M2	140299	140336	reverse	38
M1	102079	102116	M2	140299	140336	forward	38
M1	405245	405282	M2	67244	67281	forward	38
M1	536750	536787	M2	70344	70381	forward	38

M1	463697	463733	M2	75668	75704	reverse	37
M1	158355	158390	M2	6080	6115	reverse	36
M1	223695	223730	M2	153239	153274	reverse	36
M1	280325	280360	M2	114181	114216	reverse	36
M1	405201	405236	M2	67200	67235	forward	36
M1	528206	528241	M2	153239	153274	reverse	36
M1	58758	58792	M2	143354	143388	forward	35
M1	83483	83517	M2	140447	140481	reverse	35
M1	186364	186398	M2	82536	82570	reverse	35
M1	193827	193861	M2	7835	7869	forward	35
M1	426356	426390	M2	84363	84397	forward	35
M1	59259	59292	M2	76501	76534	reverse	34
M1	515807	515840	M2	3637	3670	reverse	34
M1	538562	538595	M2	28671	28704	reverse	34
M1	7917	7949	M2	26709	26741	reverse	33
M1	17483	17515	M2	114184	114216	reverse	33
M1	60221	60253	M2	75543	75575	reverse	33
M1	75042	75074	M2	52842	52874	reverse	33
M1	167472	167504	M2	52842	52874	forward	33
M1	215038	215070	M2	114602	114634	forward	33
M1	269208	269240	M2	26709	26741	forward	33
M1	358929	358961	M2	114184	114216	reverse	33
M1	399325	399357	M2	26709	26741	forward	33
M1	537648	537680	M2	11357	11389	forward	33
M1	75041	75072	M2	33203	33234	forward	32
M1	117790	117821	M2	10871	10902	forward	32
M1	243019	243050	M2	77890	77921	forward	32
M1	475999	476030	M2	61604	61635	forward	32
M1	552936	552967	M2	154288	154319	reverse	32
M1	17705	17735	M2	116566	116596	forward	31
M1	28243	28273	M2	140262	140292	reverse	31
M1	167474	167504	M2	33204	33234	reverse	31
M1	86444	86473	M2	7639	7668	forward	30
M1	293455	293484	M2	111434	111463	forward	30
M1	381099	381128	M2	5417	5446	reverse	30

Repeats M2-M2

M2	157915	158155	M2	158212	158452	forward	241
M2	70666	70731	M2	70795	70860	forward	66
M2	11408	11455	M2	12191	12238	forward	48
M2	54037	54084	M2	145435	145482	reverse	48
M2	11357	11403	M2	12140	12186	forward	47
M2	107621	107661	M2	142058	142098	reverse	41
M2	138041	138079	M2	151589	151627	reverse	39
M2	33204	33234	M2	52844	52874	reverse	31
M2	158155	158185	M2	158450	158480	forward	31

Table S8: Tandem repeats in *Ludwigia grandiflora* subsp. *hexapetala* and *Ludwigia peploides* subsp. *montevidensis* mitogenomes.

Ludwigia grandiflora* subsp. *hexapetala			
**Coordinates**	**Period size**	**Copy number**	**Percent matches**
**Molecule M2**			
146,440-146,485	22	2.1	100
**Molecule M1**			
18,305-18,347	18	2.4	81
44,204-44,240	17	2.2	90
44,754-44,787	17	2.0	88
62,384-62,413	15	2.0	100
96,281-96,313	17	1.9	93
127,742-127,424	18	2.4	81
154,823-154,847	12	2.1	100
170,080-170,113	15	2.3	94
171,929-172,009	41	2.0	90
225,692-225,734	11	3.9	93
251,276-251,316	21	1.9	90
287,158-287,206	21	3.9	70
287,208-287,249	18	2.0	100
287,208-287,249	21	2.0	95
296,977-297,053	24	3.5	74
296,993-297,033	18	2.3	95
314,519-314,556	16	2.4	91
332,357-332,406	25	2.0	100
382,053-382,089	19	1.9	88
387,587-387,629	18	2.4	81
438,208-438,268	9	6.8	94
482,654-482,687	16	2.1	94
514,004-514,082	39	2.0	95
519,531-519,564	15	2.3	94
Ludwigia peploides* subsp. *montevidensis			
**Molecule M2**			
146,342-146,387	22	2.1	100
157,911-158,505	297	2.0	98
**Molecule M1**			
8,683-8,725	18	2.4	81
14,223-14,259	19	1.9	88
63,874-63,923	25	2.0	100
81,700-81,737	16	2.4	91
100,869-100,932	18	3.6	91
144,651-144,691	21	1.9	90
170,230-170,272	11	3.9	93
225,782-225,815	15	2.3	94
241,032-241,056	12	2.1	100
268,432-268,474	18	2.4	81
319,773-319,833	9	6.8	98
372,671-372,707	17	2.2	90
398,549-398,591	18	2.4	81
493,434-493,467	16	2.1	94
501,801-501,830	15	2.0	100
524,767-524,845	39	2.0	95
530,293-530,326	15	2.3	94

Table S9: The relative synonymous codon usage (RSCU) bias in *Ludwigia grandiflora* subsp. *hexapetala* and *Ludwigia peploides* subsp. *montevidensis* mitogenomes CDS (Sequences of protein coding genes).

Ludwigia grandiflora* subsp. *hexapetala

**Codon**	**Aminoacid**	**Count**	**RSCU**
GCA	Ala	200	1.09
GCC	Ala	169	0.92
GCG	Ala	79	0.43
GCU	Ala	288	1.57
AGA	Arg	204	1.47
AGG	Arg	111	0.8
CGA	Arg	189	1.36
CGC	Arg	72	0.52
CGG	Arg	99	0.71
CGU	Arg	160	1.15
AAC	Asn	122	0.66
AAU	Asn	247	1.34
GAC	Asp	110	0.6
GAU	Asp	256	1.4
UGC	Cys	76	0.86
UGU	Cys	100	1.14
UAA	End	23	1.64
UAG	End	4	0.29
UGA	End	15	1.07
CAA	Gln	235	1.52
CAG	Gln	75	0.48
GAA	Glu	332	1.35
GAG	Glu	160	0.65
GGA	Gly	307	1.51
GGC	Gly	108	0.53
GGG	Gly	144	0.71
GGU	Gly	256	1.26
CAC	His	68	0.48
CAU	His	216	1.52
AUA	Ile	246	0.84
AUC	Ile	252	0.86
AUU	Ile	385	1.31
CUA	Leu	176	0.87
CUC	Leu	131	0.64
CUG	Leu	120	0.59
CUU	Leu	266	1.31
UUA	Leu	275	1.35
UUG	Leu	251	1.24
AAA	Lys	325	1.18
AAG	Lys	225	0.82
AUG	Met	302	1
UUC	Phe	311	0.86
UUU	Phe	410	1.14
CCA	Pro	167	1.09
CCC	Pro	121	0.79
CCG	Pro	99	0.65
CCU	Pro	224	1.47
AGC	Ser	110	0.61
AGU	Ser	186	1.04
UCA	Ser	209	1.17
UCC	Ser	178	0.99
UCG	Ser	148	0.83
UCU	Ser	245	1.37
ACA	Thr	140	0.97
ACC	Thr	141	0.98
ACG	Thr	84	0.58
ACU	Thr	213	1.47
UGG	Trp	174	1
UAC	Tyr	82	0.48
UAU	Tyr	261	1.52
GUA	Val	215	1.21
GUC	Val	133	0.75
GUG	Val	155	0.87
GUU	Val	210	1.18

Ludwigia peploides* subsp. *montevidensis

**Codon**	**Aminoacid**	**Count**	**RSCU**
GCA	Ala	219	1.13
GCC	Ala	176	0.9
GCG	Ala	81	0.42
GCU	Ala	302	1.55
AGA	Arg	212	1.46
AGG	Arg	117	0.81
CGA	Arg	196	1.35
CGC	Arg	71	0.49
CGG	Arg	105	0.72
CGU	Arg	169	1.17
AAC	Asn	127	0.64
AAU	Asn	272	1.36
GAC	Asp	117	0.6
GAU	Asp	273	1.4
UGC	Cys	77	0.84
UGU	Cys	107	1.16
UAA	End	24	1.6
UAG	End	7	0.47
UGA	End	14	0.93
CAA	Gln	248	1.51
CAG	Gln	81	0.49
GAA	Glu	360	1.37
GAG	Glu	166	0.63
GGA	Gly	321	1.51
GGC	Gly	110	0.52
GGG	Gly	150	0.71
GGU	Gly	268	1.26
CAC	His	70	0.48
CAU	His	223	1.52
AUA	Ile	257	0.83
AUC	Ile	263	0.85
AUU	Ile	410	1.32
CUA	Leu	181	0.85
CUC	Leu	133	0.62
CUG	Leu	123	0.58
CUU	Leu	280	1.31
UUA	Leu	301	1.41
UUG	Leu	263	1.23
AAA	Lys	343	1.19
AAG	Lys	234	0.81
AUG	Met	316	1
UUC	Phe	318	0.85
UUU	Phe	428	1.15
CCA	Pro	175	1.12
CCC	Pro	122	0.78
CCG	Pro	101	0.65
CCU	Pro	228	1.46
AGC	Ser	112	0.61
AGU	Ser	193	1.05
UCA	Ser	214	1.16
UCC	Ser	183	0.99
UCG	Ser	151	0.82
UCU	Ser	251	1.36
ACA	Thr	148	0.97
ACC	Thr	147	0.97
ACG	Thr	91	0.6
ACU	Thr	222	1.46
UGG	Trp	187	1
UAC	Tyr	83	0.46
UAU	Tyr	277	1.54
GUA	Val	224	1.21
GUC	Val	133	0.72
GUG	Val	160	0.86
GUU	Val	226	1.22

Table S10: Pairwise comparisons of non-synonymous substitution rate (Ka) and synonymous substitution rate (Ks) ratios of Myrtales and Geraniales order species with *Ludwigia grandiflora* subsp. *hexapetala* and *Ludwigia peploides* subsp. *montevidensis* for 31 mitochondrial genes. Genes present in at least 14 of the 16 selected species were studied. Values in blue are >1.

Genes	*O. villaricae*	*O. biennis*	*O. elata*	*C. angustifolium*	*L. indica*	*P. granatum*	*M. candidum*	*M. sanguineum*	*M. dodecandrum*	*S. samarangense*	*R. tomentosa*	*E. urophylla X grandis*	*E. grandis*	*G. maderenese*
nad1	0.15	0.15	0.15	0.15	0.19	0.17	0.29	0.29	0.29	0.45	0.55	0.45	0.45	0.33
nad2	0.22	0.22	0.22	0.16	0.45	0.37	0.26	0.26	0.27	0.28	0.24	0.19	0.25	0.51
nad3	1.59	1.59	1.59	0.95	0.63	0.79	0.87	0.87	0.81	0.18	NA	0.24	0.18	0.36
nad4	0.28	0.28	0.28	0.33	0.15	0.15	0.15	0.15	0.17	0.27	0.27	0.3	0.26	0.79
nad4L	0.21	0.21	0.21	0.32	0.24	0.24	0.15	0.15	0.15	0.15	0.15	NA	0.15	0.17
nad5	0.29	0.29	0.29	0.35	0.3	0.24	0.19	0.19	0.23	0.36	0.32	0.32	0.35	0.44
nad6	0.09	0.11	0.09	0.11	0.17	0.17	0.18	0.18	0.17	0.09	0.09	0.09	0.09	0.37
nad7	0.09	0.09	0.09	0.13	0.24	0.1	0.31	0.31	0.31	0.16	NA	0.27	0.37	0.41
nad9	0.3	0.3	0.3	0.45	0.45	0.45	0.5	0.5	0.5	0.45	0.3	0.3	1.26	0.48
sdh4	0.48	0.48	0.48	0.51	0.45	0.46	0.44	0.44	0.44	0.5	0.5	0.47	0.47	NA
cob	0.24	0.24	0.24	0.25	0.14	0.13	0.2	0.2	0.2	0.22	0.18	0.2	0.22	0.23
cox1	0.17	0.18	0.18	0.21	0.19	0.2	0.28	0.28	0.28	0.23	0.21	0.22	0.23	0.16
cox2	0.28	0.31	0.31	0.34	0.3	0.25	0.3	0.3	0.3	0.34	NA	0.37	0.3	0.31
cox3	0.06	0.06	0.06	0.08	0.37	0.23	0.2	0.2	0.2	0.26	0.26	0.29	0.23	0.27
atp1	0.12	0.12	0.12	0.12	0.16	0.13	0.28	0.28	0.28	0.13	NA	0.13	0.12	0.2
atp4	0.46	0.46	0.46	0.49	0.44	0.55	0.36	0.36	0.36	0.47	0.48	0.52	0.48	0.51
atp6	0.26	0.28	0.28	0.28	0.28	0.35	0.45	0.45	0.45	0.42	0.45	0.46	0.46	0.25
atp8	0.13	0.07	0.07	0.28	0.46	0.46	0.13	0.13	0.15	0.15	0.33	0.22	0.22	0.45
atp9	0.04	0.04	0.04	0.04	0.33	0.33	0.1	0.1	0.1	0.18	NA	0.18	0.18	0.05
ccmB	0.38	0.38	0.38	1.01	1.45	1.2	0.42	0.42	0.42	0.5	1.01	1.01	1.01	1.04
ccmC	0.55	0.55	0.55	0.55	0.67	0.67	0.27	0.27	0.27	0.47	0.47	0.47	0.47	1.03
ccmFc	0.69	0.79	0.79	0.65	0.89	0.67	0.8	0.8	0.75	1.02	0.86	1	1	0.82
matR	0.75	0.76	0.76	0.76	0.5	0.46	0.74	0.74	0.76	0.69	NA	0.78	0.78	0.76
mttB	1.01	0.92	0.92	1.7	0.62	0.58	0.74	0.74	0.74	1.09	NA	1.16	1.16	0.68
rpl2	0.47	0.51	0.51	0.32	0.63	0.64	0.44	0.44	0.46	0.68	0.78	0.65	0.72	NA
rpl5	0.21	0.24	0.24	0.79	0.38	0.57	0.75	0.75	0.63	0.17	0.28	0.17	0.2	NA
rpl10	0.49	0.64	0.64	0.64	0.49	0.56	1.22	1.22	1.07	0.48	0.46	0.55	0.55	0.98
rps1	2.26	2.26	2.26	1.09	0.98	0.98	1.66	1.66	1.66	2.59	NA	2.61	2.61	1.53
rps3	1.32	1.32	1.32	0.83	0.75	0.57	0.59	0.59	0.59	0.59	NA	0.55	0.57	0.65
rps4	0.42	0.41	0.41	0.41	0.58	0.63	0.52	0.52	0.5	0.49	0.62	0.52	0.52	NA
rps14	0.22	0.22	0.22	0.11	0.33	0.33	0.32	0.32	0.32	0.22	0.22	0.22	0.22	NA
